# Supplementary material for: Improving the mental health and mental health support available to adolescents with social care-experience via low-intensity life story work: a realist review protocol
Source: BMJ Open. 2022 Mar 9;12(3):e058424. doi: 10.1136/bmjopen-2021-058424 (PMC8915363; doi:10.1136/bmjopen-2021-058424)
Supplement: Supplementary data [file bmjopen-2021-058424supp001.pdf]

Improving the mental health and mental health support available to adolescents with social care-experience via low-intensity life story work: A realist review protocol

Supplementary File

Search #1 Life Story Work

MEDLINE

Medline (Ovid MEDLINE® Epub Ahead of Print, In-Process & Other Non-Indexed Citations, Ovid MEDLINE® Daily and Ovid MEDLINE®) 1946 to present

Search run on 1 December 2021

	Searches	Results	Type
**1**	((life story or life history) adj2 (work or book* or resource* or tool*)).ti,ab,kw.	107	
**2**	(personal history or biograph* or autobiograph*).ti,ab,kw.	30152	
**3**	1 or 2	30262	
**4**	(teen* or youth* or adolescen* or juvenile* or young* or child* or girl? or boy?).ti,ab,kw.	2435378	
**5**	Adolescent/	2143136	
**6**	4 or 5	3811906	
**7**	looked-after.ti,ab,kw.	569	
**8**	in care.ti,ab,kw.	18400	
**9**	((foster* or social or public or state or local authority or residential or institutional or permanent or kinship or relative or substitute or out-of-home or shelter or surrogate) adj1 care*).ti,ab,kw.	19263	
**10**	(care leaver* or care-experience* or leaving care or care transition*).ti,ab,kw.	6579	
**11**	child welfare.ti,ab,kw.	5173	
**12**	(adopted or adoption or adoptive or adoptee*).ti,ab,kw.	197909	
**13**	Adoption/	4869	
**14**	Foster Home Care/	3758	
**15**	or/7-14	246890	
**16**	3 and 6 and 15	87	

Hammond SP, *et al. BMJ Open* 2022; 12:e058424. doi: 10.1136/bmjopen-2021-058424

Improving the mental health and mental health support available to adolescents with social care-experience via low-intensity life story work: A realist review protocol

Embase

Ovid Embase 1974 to Present

	Searches	Results	Type
**1**	((life story or life history) adj2 (work or book* or resource* or tool*)).ti,ab,kw.	148	
**2**	(personal history or biograph* or autobiograph*).ti,ab,kw.	25170	
**3**	1 or 2	25307	
**4**	(teen* or youth* or adolescen* or juvenile* or young* or child* or girl? or boy?).ti,ab,kw.	3050210	
**5**	adolescent/ or exp institutionalized adolescent/	1629539	
**6**	4 or 5	3924528	
**7**	looked-after.ti,ab,kw.	1002	
**8**	in care.ti,ab,kw.	26487	
**9**	((foster* or social or public or state or local authority or residential or institutional or permanent or kinship or relative or substitute or out-of-home or shelter or surrogate) adj1 care*).ti,ab,kw.	23880	
**10**	(care leaver* or care-experience* or leaving care or care transition*).ti,ab,kw.	8952	
**11**	child welfare.ti,ab,kw.	3648	
**12**	(adopted or adoption or adoptive or adoptee*).ti,ab,kw.	257330	
**13**	adoption/	18302	
**14**	foster care/ or foster child/	5008	
**15**	or/7-14	320109	
**16**	3 and 6 and 15	116	

Search run on 1 December 2021

Hammond SP, *et al. BMJ Open* 2022; 12:e058424. doi: 10.1136/bmjopen-2021-058424

Improving the mental health and mental health support available to adolescents with social care-experience via low-intensity life story work: A realist review protocol

PsycINFO

Ovid PsycINFO 1806 to Present

Search run on 1 December 2021

	Searches	Results	Type
**1**	((life story or life history) adj2 (work or book* or resource* or tool*)).ti,ab.	126	
**2**	(personal history or biograph* or autobiograph*).ti,ab.	27009	
**3**	life review/ or autobiographical memory/	5007	
**4**	or/1-3	28393	
**5**	(teen* or youth* or adolescen* or juvenile* or young* or child* or girl? or boy?).ti,ab.	1104726	
**6**	looked-after.ti,ab.	656	
**7**	in care.ti,ab.	7188	
**8**	((foster* or social or public or state or local authority or residential or institutional or permanent or kinship or relative or substitute or out-of-home or shelter or surrogate) adj1 care*).ti,ab.	19519	
**9**	(care leaver* or care-experience* or leaving care or care transition*).ti,ab.	2950	
**10**	child welfare.ti,ab.	7784	
**11**	(adopted or adoption or adoptive or adoptee*).ti,ab.	66430	
**12**	adoption (child)/ or adopted children/ or adoptive parents/	5319	
**13**	foster care/ or foster children/ or foster parents/	7544	
**14**	or/6-13	99727	
**15**	4 and 5 and 14	203	

Hammond SP, *et al. BMJ Open* 2022; 12:e058424. doi: 10.1136/bmjopen-2021-058424

Improving the mental health and mental health support available to adolescents with social care-experience via low-intensity life story work: A realist review protocol

Proquest Sociology Collection

ASSIA (Applied Social Sciences Index & Abstracts) (1987 to present); Sociological Abstracts (1952 to present); Sociology Database (1985 to present)

Search run on 1 December 2021

	Searches	Results	Type
**1**	noft(("life story" OR "life history") N/2 (work OR book* OR resource* OR tool*))	223	
**2**	noft(("personal history" OR biograph* OR autobiograph*))	30,652	
**3**	1 or 2	30,836	
**4**	noft((teen* OR youth* OR adolescen* OR juvenile* OR young* OR child* OR boy* OR girl*))	776,851	
**5**	noft("looked after" OR "looked-after")	1,477	
**6**	noft("in care")	8,932	
**7**	noft((foster* OR social OR public OR state OR "local authority" OR residential OR institutional OR permanent OR kinship OR relative OR substitute OR "out of home" OR out-of-home OR shelter OR surrogate) N/1 care*)	49,937	
**8**	noft("care leaver" OR "care experience*" OR care-experience* OR "leaving care" OR "care transition*")	3,219	
**9**	noft("child welfare")	27,228	
**10**	noft(adopted OR adoption OR adoptive OR adoptee*)	35,937	
**11**	5 or 6 or 7 or 8 or 9 or 10	110774	
**12**	3 and 4 and 12	372	
**Full**	(noft(("life story" OR "life history") NEAR/2 (work OR book* OR resource* OR tool*)) OR noft(("personal history" OR biograph* OR autobiograph*))) AND (noft("looked after" OR "looked-after") OR noft("in care") OR noft((foster* OR social OR public OR state OR "local authority" OR residential OR institutional OR permanent OR kinship OR relative OR substitute OR "out of home" OR out-of-home OR shelter OR surrogate) NEAR/1 care*) OR noft("care leaver" OR "care experience*" OR care-experience* OR "leaving care" OR "care transition*") OR noft("child welfare") OR noft(adopted OR adoption OR adoptive OR adoptee*)) AND noft((teen* OR youth* OR adolescen* OR juvenile* OR young* OR child* OR boy* OR girl*))		

Hammond SP, *et al. BMJ Open* 2022; 12:e058424. doi: 10.1136/bmjopen-2021-058424

Improving the mental health and mental health support available to adolescents with social care-experience via low-intensity life story work: A realist review protocol

CINAHL

Ebscohost CINAHL (Cumulative Index to Nursing and Allied Health Literature) (Start date unknown)

Search run 1 December 2021

	Searches	Results	Type
**S1**	TI ("life story" OR "life history") N2 (work OR book* OR resource* OR tool*) OR AB ("life story" OR "life history") N2 (work OR book* OR resource* OR tool*)	135	
**S2**	TI ("personal history" OR biograph* OR autobiograph*) OR AB ("personal history" OR biograph* OR autobiograph*)	6148	
**S3**	(MH "Life History Review") OR (MH "Autobiographical Memory")	1592	
**S4**	S1 OR S2 OR S3	7675	
**S5**	TI teen* OR youth* OR adolescen* OR juvenile* OR young* OR child* OR girl* OR boy* OR AB teen* OR youth* OR adolescen* OR juvenile* OR young* OR child* OR girl* OR boy*	1475230	
**S6**	(MH "Adolescence")	564984	
**S7**	S5 OR S6	1475230	
**S8**	TI "looked after" OR looked-after OR AB "looked after" OR looked-after	718	
**S9**	TI "in care" OR AB "in care"	929827	
**S10**	TI (foster* OR social OR public OR state OR "local authority" OR residential OR institutional OR permanent OR kinship OR relative OR substitute OR "out of home" OR out-of-home OR shelter OR surrogate) N1 care* OR AB (foster* OR social OR public OR state OR "local authority" OR residential OR institutional OR permanent OR kinship OR relative OR substitute OR "out of home" OR out-of-home OR shelter OR surrogate) N1 care*	31046	
**S11**	TI ("care leaver" OR "care experience*" OR care-experience* OR "leaving care" OR "care transition*") OR AB ("care leaver" OR "care experience*" OR care-experience* OR "leaving care" OR "care transition*")	5417	
**S12**	TI "child welfare" OR AB "child welfare"	4202	
**S13**	TI adopted or adoption or adoptive or adoptee* OR AB adopted or adoption or adoptive or adoptee*	57750	
**S14**	(MH "Adoption") OR (MH "Child, Adopted") OR (MH "Adoptive Parents")	4085	
**S15**	(MH "Foster Home Care") OR (MH "Foster Parents") OR (MH "Child, Foster")	6372	
**S16**	S8 OR S9 OR S10 OR S11 OR S12 OR S13 OR S14 OR S15	9 75840	
**S17**	S4 AND S7 AND S16	359	

Hammond SP, *et al. BMJ Open* 2022; 12:e058424. doi: 10.1136/bmjopen-2021-058424

Improving the mental health and mental health support available to adolescents with social care-experience via low-intensity life story work: A realist review protocol

CDAS

Ebscohost CDAS (Child Development and Adolescent Studies) (Start date unknown)

Search run 1 December 2021

	Searches	Results	Type
**S1**	TI ("life story" OR "life history") N2 (work OR book* OR resource* OR tool*) OR AB ("life story" OR "life history") N2 (work OR book* OR resource* OR tool*)	29	
**S2**	TI ("personal history" OR biograph* OR autobiograph*) OR AB ("personal history" OR biograph* OR autobiograph*)	1442	
**S3**	S1 OR S2	1471	
**S4**	TI "looked after" OR looked-after OR AB "looked after" OR looked-after	696	
**S5**	TI "in care" OR AB "in care"	32026	
**S6**	TI (foster* OR social OR public OR state OR "local authority" OR residential OR institutional OR permanent OR kinship OR relative OR substitute OR "out of home" OR out-of-home OR shelter OR surrogate) N1 care* OR AB (foster* OR social OR public OR state OR "local authority" OR residential OR institutional OR permanent OR kinship OR relative OR substitute OR "out of home" OR out-of-home OR shelter OR surrogate) N1 care*	7379	
**S7**	TI ("care leaver" OR "care experience*" OR care-experience* OR "leaving care" OR "care transition*") OR AB ("care leaver" OR "care experience*" OR care-experience* OR "leaving care" OR "care transition*")	651	
**S8**	TI "child welfare" OR AB "child welfare"	5715	
**S9**	TI adopted or adoption or adoptive or adoptee* OR AB adopted or adoption or adoptive or adoptee*	6798	
**S10**	S4 OR S5 OR S6 OR S7 OR S8 OR S9	41463	
**S11**	S3 AND S10	86	
	NB Search string describing adolescents removed due to population focus in this database		

Hammond SP, *et al. BMJ Open* 2022; 12:e058424. doi: 10.1136/bmjopen-2021-058424

Improving the mental health and mental health support available to adolescents with social care-experience via low-intensity life story work: A realist review protocol

Web of Science

Clarivate Web of Science Core Collection (SCI-EXPANDED and SSCI indexes) 1900 to present

Search run 1 December 2021

	Searches	Results	Type
**1**	TS=((("life story" OR "life history") NEAR/2 (work OR book* OR resource* OR tool*)))	449	
**2**	TS=((("personal history" OR biograph* OR autobiograph*)))	41850	
**3**	1 OR 2	41850	
**4**	TS=(((teen* OR youth* OR adolescen* OR juvenile* OR young* OR child* OR boy* OR girl*)))	3066217	
**5**	TS=(("looked after" OR "looked-after"))	768	
**6**	TS=(("in care"))	16889	
**7**	TS=((foster* OR social OR public OR state OR "local authority" OR residential OR institutional OR permanent OR kinship OR relative OR substitute OR "out of home" OR out-of-home OR shelter OR surrogate) NEAR/1 care*)	43040	
**8**	TS=("care leaver" OR "care experience*" OR care-experience* OR "leaving care" OR "care transition*")	6615	
**9**	TS=("child welfare")	8348	
**10**	TS=(adopted OR adoption OR adoptive OR adoptee*)	655351	
**11**	5 OR 6 OR 7 OR 8 OR 9 OR 10	721923	
**12**	3 AND 4 AND 11	233	

Hammond SP, *et al. BMJ Open* 2022; 12:e058424. doi: 10.1136/bmjopen-2021-058424

Improving the mental health and mental health support available to adolescents with social care-experience via low-intensity life story work: A realist review protocol

Social Care Online

SCIE Social Care Online 1980 to present

Search run 3 February 2022

	Searches	Results	Type
**1**	“life story” OR “life history”		
**2**	teen OR youth OR adolescent OR juvenile OR young OR child OR girl OR boy		
**3**	“looked after” OR “foster care” OR “social care” OR “public care” OR “state care” OR “local authority care” OR “residential care” OR "institutional care" OR "permanent care" OR "kinship care" OR "relative care" OR "substitute care" OR "out of home care" OR "shelter care" OR adopted OR adoption OR adoptive OR adoptee		
**4**	(1 AND 2 AND 3)	58	
	NB Search strategies were adapted for simpler search interface; word variations included automatically		

Hammond SP, *et al. BMJ Open* 2022; 12:e058424. doi: 10.1136/bmjopen-2021-058424

Improving the mental health and mental health support available to adolescents with social care-experience via low-intensity life story work: A realist review protocol

Search #2 Low intensity mental health interventions

MEDLINE

Medline (Ovid MEDLINE® Epub Ahead of Print, In-Process & Other Non-Indexed Citations, Ovid MEDLINE® Daily and Ovid MEDLINE®) 1946 to present

Search run on 2 February 2022

	Searches	Results	Type
1	(teen* or youth* or adolescen* or juvenile*).ti,ab,kw.	486326	
2	Adolescent/	2155772	
3	1 or 2	2326981	
4	looked-after.ti,ab,kw.	585	
5	((foster* or social or public or state or local authority or residential or institutional or permanent or kinship or relative or substitute or out-of-home or shelter or surrogate) adj1 care*).ti,ab,kw.	19561	
6	(care leaver* or care-experience* or leaving care or care transition*).ti,ab,kw.	6715	
7	child welfare.ti,ab,kw.	5217	
8	Adoption/	4875	
9	Foster Home Care/	3770	
10	or/4-9	37119	
11	((adopted or adoption or adoptive or adoptee* or in-care or "in care") adj3 (teen* or youth* or adolescen* or juvenile* or young* or child* or girl? or boy?)).ti,ab,kw.	3708	
12	10 or 11	38990	
13	((low-intensity or minimal or brief or online or internet or tele* or mobile* or e-health or m-health or virtual) adj2 (intervention* or program* or service* or package* or training* or therap* or treatment*)).ti,ab,kw.	48784	
14	Therapy, Computer-Assisted/	6949	
15	Computer-Assisted Instruction/	12318	
16	Telemedicine/	32176	
17	or/13-16	92385	
18	(lay-therapy or lay-therapist* or lay-worker* or lay-person* or lay-people).ti,ab,kw.	2143	
19	(para-professional* or non-specialist* or non-clinician* or health worker* or support worker*).ti,ab,kw.	24797	
20	((unqualified or unregistered or volunt*) adj2 (therap* or worker* or coach* or facilitator* or practitioner*)).ti,ab,kw.	1337	
21	Community Health Workers/	6092	
22	or/18-21	30823	
23	(self-help or self-manage*).ti,ab,kw.	31158	
24	Self Care/	35033	
25	or/23-24	57185	
26	((group* or peer*) adj2 (intervention* or program* or service* or package* or training* or therap* or treatment)).ti,ab,kw.	228457	

Hammond SP, et al. BMJ Open 2022; 12:e058424. doi: 10.1136/bmjopen-2021-058424

Improving the mental health and mental health support available to adolescents with social care-experience via low-intensity life story work: A realist review protocol

**27**	Self-Help Groups/	9435
**28**	or/26-27	236909
**29**	17 or 22 or 25 or 28	398896
**30**	(mental health or wellbeing or well-being).ti,ab,kw.	280239
**31**	mental health/ or exp mental disorders/	1382592
**32**	Quality of Life/	232462
**33**	or/30-32	1732433
**34**	3 and 12 and 29 and 33	160
**35**	limit 34 to english language	151

Hammond SP, *et al. BMJ Open* 2022; 12:e058424. doi: 10.1136/bmjopen-2021-058424

Improving the mental health and mental health support available to adolescents with social care-experience via low-intensity life story work: A realist review protocol

Embase

Ovid Embase 1974 to Present

Search run on 2 February 2022

	Searches	Results	Type
1	(teen* or youth* or adolescen* or juvenile*).ti,ab,kw.	601244	
2	adolescent/ or institutionalized adolescent/	1644483	
3	1 or 2	1868684	
4	looked-after.ti,ab,kw.	1012	
5	((foster* or social or public or state or local authority or residential or institutional or permanent or kinship or relative or substitute or out-of-home or shelter or surrogate) adj1 care*).ti,ab,kw.	24293	
6	(care leaver* or care-experience* or leaving care or care transition*).ti,ab,kw.	9146	
7	child welfare.ti,ab,kw.	3695	
8	adoption/	18546	
9	foster care/ or foster child/	5037	
10	or/4-9	56946	
11	((adopted or adoption or adoptive or adoptee* or in-care or "in care") adj3 (teen* or youth* or adolescen* or juvenile* or young* or child* or girl? or boy?)).ti,ab,kw.	4485	
12	10 or 11	59432	
13	((low-intensity or minimal or brief or online or internet or tele* or mobile* or e-health or m-health or virtual) adj2 (intervention* or program* or service* or package* or training* or therap* or treatment*)).ti,ab,kw.	65233	
14	computer assisted therapy/	4801	
15	Computer-Assisted Instruction/	81963	
16	telemedicine/ or exp teleconsultation/ or exp telemonitoring/ or exp telepsychiatry/ or exp telepsychology/ or exp teletherapy/ or exp video consultation/	51669	
17	or/13-16	193129	
18	(lay-therapy or lay-therapist* or lay-worker* or lay-person* or lay-people).ti,ab,kw.	2836	
19	(para-professional* or non-specialist* or non-clinician* or health worker* or support worker*).ti,ab,kw.	29728	
20	((unqualified or unregistered or volunt*) adj2 (therap* or worker* or coach* or facilitator* or practitioner*)).ti,ab,kw.	1615	
21	voluntary worker/ or exp volunteer/	64329	
22	lay health worker/ or mental health care personnel/ or health auxiliary/	11904	
23	or/18-22	103668	
24	(self-help or self-manage*).ti,ab,kw.	43320	

Hammond SP, *et al. BMJ Open* 2022; 12:e058424. doi: 10.1136/bmjopen-2021-058424

Improving the mental health and mental health support available to adolescents with social care-experience via low-intensity life story work: A realist review protocol

**25**	self care/ or self help/	80302
**26**	or/24-25	94122
**27**	((group* or peer*) adj2 (intervention* or program* or service* or package* or training* or therap* or treatment)).ti,ab,kw.	333041
**28**	group therapy/	19771
**29**	or/27-28	343422
**30**	17 or 23 or 26 or 29	706680
**31**	(mental health or wellbeing or well-being).ti,ab,kw.	349720
**32**	exp mental health/ or exp mental disease/	2482186
**33**	quality of life/ or exp wellbeing/	614976
**34**	or/31-33	3026040
**35**	3 and 12 and 30 and 34	283
**36**	limit 35 to english language	274

Hammond SP, *et al. BMJ Open* 2022; 12:e058424. doi: 10.1136/bmjopen-2021-058424

Improving the mental health and mental health support available to adolescents with social care-experience via low-intensity life story work: A realist review protocol

PsycINFO

Ovid PsycINFO 1806 to Present

Search run on 2 February 2022

	Searches	Results	Type
**1**	(teen* or youth* or adolescen* or juvenile*).ti,ab.	344737	
**2**	looked-after.ti,ab.	662	
**3**	((foster* or social or public or state or local authority or residential or institutional or permanent or kinship or relative or substitute or out-of-home or shelter or surrogate) adj1 care*).ti,ab.	19672	
**4**	(care leaver* or care-experience* or leaving care or care transition*).ti,ab.	2980	
**5**	child welfare.ti,ab.	7836	
**6**	adoption (child)/ or adopted children/ or adoptive parents/	5337	
**7**	foster care/ or foster children/ or foster parents/	7582	
**8**	or/2-7	34369	
**9**	((adopted or adoption or adoptive or adoptee* or in-care or "in care") adj3 (teen* or youth* or adolescen* or juvenile* or young* or child* or girl? or boy?)).ti,ab.	5753	
**10**	8 or 9	36284	
**11**	((low-intensity or minimal or brief or online or internet or tele* or mobile* or e-health or m-health or virtual) adj2 (intervention* or program* or service* or package* or training* or therap* or treatment*)).ti,ab.	28828	
**12**	exp computer assisted therapy/	11642	
**13**	exp computer assisted instruction/	21848	
**14**	or/11-13	57608	
**15**	(lay-therapy or lay-therapist* or lay-worker* or lay-person* or lay-people).ti,ab.	1793	
**16**	(para-professional* or non-specialist* or non-clinician* or health worker* or support worker*).ti,ab.	7045	
**17**	((unqualified or unregistered or volunt*) adj2 (therap* or worker* or coach* or facilitator* or practitioner*)).ti,ab.	746	
**18**	volunteers/	5507	
**19**	or/15-18	14762	
**20**	(self-help or self-manage*).ti,ab.	19273	
**21**	self-care/	3097	
**22**	self-help techniques/	4398	
**23**	or/20-22	23360	

Hammond SP, *et al. BMJ Open* 2022; 12:e058424. doi: 10.1136/bmjopen-2021-058424

Improving the mental health and mental health support available to adolescents with social care-experience via low-intensity life story work: A realist review protocol

**24**	((group* or peer*) adj2 (intervention* or program* or service* or package* or training* or therap* or treatment)).ti,ab.	75638
**25**	group psychotherapy/	20529
**26**	support groups/	4449
**27**	or/24-26	86800
**28**	14 or 19 or 23 or 27	173409
**29**	(mental health or wellbeing or well-being).ti,ab.	289304
**30**	exp mental health/	77446
**31**	exp mental disorders/	919736
**32**	well being/ or exp spiritual well being/	51717
**33**	quality of life/	44133
**34**	or/29-33	1164452
**35**	1 and 10 and 28 and 34	137
**36**	limit 35 to english language	130

Hammond SP, *et al. BMJ Open* 2022; 12:e058424. doi: 10.1136/bmjopen-2021-058424

Improving the mental health and mental health support available to adolescents with social care-experience via low-intensity life story work: A realist review protocol

Proquest Sociology Collection

ASSIA (Applied Social Sciences Index & Abstracts) (1987 to present); Sociological Abstracts (1952 to present); Sociology Database (1985 to present)

Search run on 2 February 2022

	Searches	Results	Type
**Full String**	(noft((teen* OR youth* OR adolescen* OR juvenile*)) AND (noft("looked after" OR "looked-after") OR noft((foster* OR social OR public OR state OR "local authority" OR residential OR institutional OR permanent OR kinship OR relative OR substitute OR "out of home" OR out-of-home OR shelter OR surrogate) N/1 care*) OR noft("care leaver" OR "care experience*" OR care-experience* OR "leaving care" OR "care transition*") OR noft("child welfare") OR noft(((adopted or adoption or adoptive or adoptee* or in-care or "in care") N/3 (teen* or youth* or adolescen* or juvenile* or young* or child* or girl? or boy?)))) AND (noft((low-intensity or minimal or brief or online or internet or tele* or mobile* or e-health or m-health or virtual) N/2 (intervention* or program* or service* or package* or training* or therap* or treatment*)) OR noft((lay-therapy or lay-therapist* or lay-worker* or lay-person* or lay-people)) OR noft((para-professional* or non-specialist* or non-clinician* or ("health worker" OR "health workers") or ("support worker" OR "support workers"))) OR noft((unqualified OR unregistered OR volunt*) NEAR/2 (therap* OR worker* OR coach* OR facilitator* OR practitioner*)) OR noft((self-help OR self-manage*)) OR noft((group* or peer*) N/2 (intervention* or program* or service* or package* or training* or therap* or treatment)))) AND noft("mental health" or wellbeing or well-being)	249	
	(Filter: English language)		

Hammond SP, *et al. BMJ Open* 2022; 12:e058424. doi: 10.1136/bmjopen-2021-058424

Improving the mental health and mental health support available to adolescents with social care-experience via low-intensity life story work: A realist review protocol

CINAHL

Ebscohost CINAHL (Cumulative Index to Nursing and Allied Health Literature) (Start date unknown)

Search run 2 February 2022

	Searches	Results	Type
**S32**	S25 AND S30	471	
**S31**	S25 AND S30	477	
**S30**	S26 OR S27 OR S28 OR S29	835,613	
**S29**	(MH "Quality of Life")	127,552	
**S28**	(MH "Mental Disorders+")	611,172	
**S27**	(MH "Mental Health")	46,077	
**S26**	TI ("mental health" OR wellbeing OR well-being) OR AB ("mental health" or wellbeing or well-being)	182,035	
**S25**	S12 AND S24	1,451	
**S24**	S13 OR S14 OR S15 OR S16 OR S17 OR S18 OR S19 OR S20 OR S21 OR S22 OR S23	216,784	
**S23**	(MH "Support Groups")	11,056	
**S22**	TI (((group* or peer*) N2 (intervention* or program* or service* or package* or training* or therap* or treatment))) OR AB (((group* or peer*) N2 (intervention* or program* or service* or package* or training* or therap* or treatment)))	99,368	
**S21**	(MH "Self Care")	42,907	
**S20**	TI ("self help" OR self-help OR "self manage*" OR self-manage*) OR AB ("self help" OR self-help OR "self manage*" OR self-manage*)	20,838	
**S19**	(MH "Community Health Workers")	4,008	
**S18**	TI (((unqualified or unregistered or volunt*) N2 (therap* or worker* or coach* or facilitator* or practitioner*))) OR AB (((unqualified or unregistered or volunt*) N2 (therap* or worker* or coach* or facilitator* or practitioner*)))	962	
**S17**	TI (("lay therapy" or lay-therapy or "lay therapist*" or lay-therapist* or "lay worker*" or lay-worker* or "lay person*" or lay-person* or "lay people" or lay-people)) AND AB (("lay therapy" or lay-therapy or "lay therapist*" or lay-	82	

Hammond SP, *et al. BMJ Open* 2022; 12:e058424. doi: 10.1136/bmjopen-2021-058424

Improving the mental health and mental health support available to adolescents with social care-experience via low-intensity life story work: A realist review protocol

	therapist* or "lay worker*" or lay-worker* or "lay person*" or lay-person* or "lay people" or lay-people))	
**S16**	(MH "Telemedicine") OR (MH "Remote Consultation") OR (MH "Telepsychiatry")	17,115
**S15**	(MH "Computer Assisted Instruction")	8,174
**S14**	(MH "Therapy, Computer Assisted")	5,457
**S13**	TI (((low-intensity or "low intensity" or minimal or brief or online or internet or tele* or mobile* or e-health or m-health or virtual) N2 (intervention* or program* or service* or package* or training* or therap* or treatment*))) OR AB (((low-intensity or "ow intensity" or minimal or brief or online or internet or tele* or mobile* or e-health or m-health or virtual) N2 (intervention* or program* or service* or package* or training* or therap* or treatment*)))	34,674
**S12**	S10 AND S11	19,442
**S11**	S3 OR S4 OR S5 OR S6 OR S7 OR S8 OR S9	77,416
**S10**	S1 OR S2	626,344
**S9**	(MH "Foster Home Care") OR (MH "Foster Parents") OR (MH "Child, Foster")	6,425
**S8**	(MH "Adoption") OR (MH "Child, Adopted") OR (MH "Adoptive Parents")	4,102
**S7**	TI (((adopted OR adoption OR adoptive OR adoptee* OR "in-care" OR "in care") N2 (teen* OR youth* OR adolescen* OR juvenile* or young* OR child*))) OR AB (((adopted OR adoption OR adoptive OR adoptee* OR "in-care" OR "in care") N2 (teen* OR youth* OR adolescen* OR juvenile* OR young* OR child*)))	37,133
**S6**	TI "child welfare" OR AB "child welfare"	4,278
**S5**	TI ("care leaver" OR "care experience*" OR care-experience* OR "leaving care" OR "care transition*") OR AB ("care leaver" OR "care experience*" OR care-experience* OR "leaving care" OR "care transition*")	5,533
**S4**	TI (foster* OR social OR public OR state OR "local authority" OR residential OR institutional OR permanent OR kinship OR relative OR substitute OR "out of home" OR out-of-home OR shelter OR surrogate) N1 care* OR AB (foster* OR social OR public OR state OR "local authority" OR residential OR institutional OR permanent OR kinship OR relative OR substitute OR "out of home" OR out-of-home OR shelter OR surrogate) N1 care*	31,528
**S3**	TI "looked after" OR looked-after OR AB "looked after" OR looked-after	723
**S2**	(MH ""Adolescence"")	569,286
**S1**	TI (teen* OR youth* OR adolescen* OR juvenile*) OR AB (teen* OR youth* OR adolescen* OR juvenile*)	209,502

Improving the mental health and mental health support available to adolescents with social care-experience via low-intensity life story work: A realist review protocol

Hammond SP, *et al. BMJ Open* 2022; 12:e058424. doi: 10.1136/bmjopen-2021-058424

Improving the mental health and mental health support available to adolescents with social care-experience via low-intensity life story work: A realist review protocol

CDAS

Ebscohost CDAS (Child Development and Adolescent Studies) (Start date unknown)

Search run 3 February 2022

	Searches	Results	Type
**S1**	TI (teen* OR youth* OR adolescen* OR juvenile*) OR AB (teen* OR youth* OR adolescen* OR juvenile*)	99,382	
**S2**	TI "looked after" OR looked-after OR AB "looked after" OR looked-after	698	
**S3**	TI (foster* OR social OR public OR state OR "local authority" OR residential OR institutional OR permanent OR kinship OR relative OR substitute OR "out of home" OR out-of-home OR shelter OR surrogate) N1 care* OR AB (foster* OR social OR public OR state OR "local authority" OR residential OR institutional OR permanent OR kinship OR relative OR substitute OR "out of home" OR out-of-home OR shelter OR surrogate) N1 care*	7434	
**S4**	TI ("care leaver" OR "care experience*" OR care-experience* OR "leaving care" OR "care transition*") OR AB ("care leaver" OR "care experience*" OR care-experience* OR "leaving care" OR "care transition*")	656	
**S5**	TI "child welfare" OR AB "child welfare"	5752	
**S6**	TI (((adopted OR adoption OR adoptive OR adoptee* OR "in-care" OR "in care") N2 (teen* OR youth* OR adolescen* OR juvenile* or young* OR child*))) OR AB (((adopted OR adoption OR adoptive OR adoptee* OR "in-care" OR "in care") N2 (teen* OR youth* OR adolescen* OR juvenile* OR young* OR child*)))	14908	
**S7**	TI (((low-intensity or "low intensity" or minimal or brief or online or internet or tele* or mobile* or e-health or m-health or virtual) N2 (intervention* or program* or service* or package* or training* or therap* or treatment*))) OR AB (((low-intensity or "ow intensity" or minimal or brief or online or internet or tele* or mobile* or e-health or m-health or virtual) N2 (intervention* or program* or service* or package* or training* or therap* or treatment*)))	2481	
**S8**	TI (("lay therapy" or lay-therapy or "lay therapist*" or lay-therapist* or "lay worker*" or lay-worker* or "lay person*" or lay-person* or "lay people" or lay-people)) AND AB (("lay therapy" or lay-therapy or "lay therapist*" or lay-therapist* or "lay worker*" or lay-worker* or "lay	5	

Hammond SP, *et al. BMJ Open* 2022; 12:e058424. doi: 10.1136/bmjopen-2021-058424

	person*" or lay-person* or "lay people" or lay-people))	
**S9**	TI (((unqualified or unregistered or volunt*) N2 (therap* or worker* or coach* or facilitator* or practitioner*))) OR AB (((unqualified or unregistered or volunt*) N2 (therap* or worker* or coach* or facilitator* or practitioner*)))	78
**S10**	TI ("self help" OR self-help OR "self manage*" OR self-manage*) OR AB ("self help" OR self-help OR "self manage*" OR self-manage*)	804
**S11**	TI (((group* or peer*) N2 (intervention* or program* or service* or package* or training* or therap* or treatment))) OR AB (((group* or peer*) N2 (intervention* or program* or service* or package* or training* or therap* or treatment)))	6798
**S12**	TI ("mental health" OR wellbeing OR well-being) OR AB ("mental health" or wellbeing or well-being)	24106
**S13**	S2 OR S3 OR S4 OR S5 OR S6	22614
**S14**	S7 OR S8 OR S9 OR S10 OR S11	9795
**S15**	S1 AND S12 AND S13 AND S14	61

Hammond SP, *et al. BMJ Open* 2022; 12:e058424. doi: 10.1136/bmjopen-2021-058424

Improving the mental health and mental health support available to adolescents with social care-experience via low-intensity life story work: A realist review protocol

Web of Science

Clarivate Web of Science Core Collection (SCI-EXPANDED and SSCI indexes) 1900 to present

Search run 2 February 2022

	Searches	Results	Type
1	TS=(((teen* OR youth* OR adolescen* OR juvenile*)))	757523	
2	TS=(("looked after" OR "looked-after"))	776	
3	TS=((foster* OR social OR public OR state OR "local authority" OR residential OR institutional OR permanent OR kinship OR relative OR substitute OR "out of home" OR out-of-home OR shelter OR surrogate) NEAR/1 care*)	43729	
4	TS=("care leaver" OR "care experience*" OR care-experience* OR "leaving care" OR "care transition*")	6760	
5	TS=("child welfare")	8425	
6	TS=(((adopted or adoption or adoptive or adoptee* or "in-care" or "in care") NEAR/3 (teen* or youth* or adolescen* or juvenile* or young* or child* or girl? or boy?)))	7120	
7	or/2-6	61902	
8	TS=(((low-intensity or minimal or brief or online or internet or tele* or mobile* or e-health or m-health or virtual) NEAR/2 (intervention* or program* or service* or package* or training* or therap* or treatment*)))	90429	
9	TS=((lay-therapy or lay-therapist* or lay-worker* or lay-person* or lay-people))	2561	
10	TS=((para-professional* or non-specialist* or non-clinician* or health worker* or support worker*))	120315	
11	TS=(((unqualified or unregistered or volunt*) NEAR/2 (therap* or worker* or coach* or facilitator* or practitioner*)))	1938	
12	TS=(self-help or self-manage*)	41426	
13	TS=((group* or peer*) NEAR/2 (intervention* or program* or service* or package* or training* or therap* or treatment))	263661	
14	#13 OR #12 OR #11 OR #10 OR #8 OR #9	499546	
15	TS=((mental health or wellbeing or well-being))	409139	
16	#15 and #14 and #7 and #1		
17	limit to english language		

Hammond SP, et al. BMJ Open 2022; 12:e058424. doi: 10.1136/bmjopen-2021-058424

Improving the mental health and mental health support available to adolescents with social care-experience via low-intensity life story work: A realist review protocol

Social Care Online

SCIE Social Care Online 1980 to present

Search run 3 February 2022

	Searches	Results	Type
**1**	teen or youth or adolescent or juvenile		
**2**	looked after OR "foster care" OR "social care" OR "public care" OR "state care" OR "local authority care" OR "residential care" OR "institutional care" OR "permanent care" OR "kinship care" OR "relative care" OR "substitute care" OR "out of home care" OR "shelter care" OR adopted OR adoption OR adoptive OR adoptee		
**3**	*low intensity OR minimal OR brief OR online OR digital OR internet OR tele OR mobile OR e-health OR m-health OR virtual*		
**4**	mental health OR wellbeing OR "well being"		
**5**	1 and 2 and 3 and 4	29	
**1**	teen or youth or adolescent or juvenile		
**2**	looked after OR "foster care" OR "social care" OR "public care" OR "state care" OR "local authority care" OR "residential care" OR "institutional care" OR "permanent care" OR "kinship care" OR "relative care" OR "substitute care" OR "out of home care" OR "shelter care" OR adopted OR adoption OR adoptive OR adoptee		
**3**	*"lay therapy" OR "lay therapist" OR "lay worker" OR "lay person" OR "lay people" OR "paraprofessional" OR "non-specialist" OR "non-clinician" OR "health worker" OR coach OR facilitator OR practitioner OR unqualified OR unregistered OR volunteer*		
**4**	mental health OR wellbeing OR "well being"		
**5**	1 and 2 and 3 and 4	7	
**1**	teen or youth or adolescent or juvenile		
**2**	looked after OR "foster care" OR "social care" OR "public care" OR "state care" OR "local authority care" OR "residential care" OR "institutional care" OR "permanent care" OR "kinship care" OR "relative care" OR "substitute care" OR "out of home care" OR "shelter care" OR adopted OR adoption OR adoptive OR adoptee		
**3**	*"self care" OR "self help" OR "self management" OR group OR peer*		
**4**	mental health OR wellbeing OR "well being"		
**5**	1 and 2 and 3 and 4	21	
	NB s*earch strategies adapted for simpler search interface and run in three separate queries*		

Hammond SP, *et al. BMJ Open* 2022; 12:e058424. doi: 10.1136/bmjopen-2021-058424

Improving the mental health and mental health support available to adolescents with social care-experience via low-intensity life story work: A realist review protocol

Hammond SP, *et al. BMJ Open* 2022; 12:e058424. doi: 10.1136/bmjopen-2021-058424
