# Supplementary material for: Improving the mental health and mental health support available to adolescents with social care-experience via low-intensity life story work: a realist review protocol
Source: BMJ Open. 2022 Mar 9;12(3):e058424. doi: 10.1136/bmjopen-2021-058424 (PMC8915363; doi:10.1136/bmjopen-2021-058424)
Supplement: Author's manuscript [file bmjopen-2021-058424.draft_revisions.pdf]

BMJ Open is committed to open peer review. As part of this commitment we make the peer review history of every article we publish publicly available.

When an article is published we post the peer reviewers' comments and the authors' responses online. We also post the versions of the paper that were used during peer review. These are the versions that the peer review comments apply to.

The versions of the paper that follow are the versions that were submitted during the peer review process. They are not the versions of record or the final published versions. They should not be cited or distributed as the published version of this manuscript.

BMJ Open is an open access journal and the full, final, typeset and author-corrected version of record of the manuscript is available on our site with no access controls, subscription charges or pay-per-view fees (http://bmjopen.bmj.com).

If you have any questions on BMJ Open's open peer review process please email info.bmjopen@bmj.com

BMJ Open

Improving the mental health and mental health support available to adolescents with social care-experience via low-intensity life story work: A realist review protocol

Journal:	*BMJ Open*
Manuscript ID	bmjopen-2021-058424
Article Type:	Protocol
Date Submitted by the Author:	19-Oct-2021
Complete List of Authors:	Hammond, Simon; University of East Anglia, School of Education and Lifelong Learning<br>Duddy, Claire; University of Oxford, Nuffield Department of Primary Care Health Sciences<br>Mickelburgh, Ella; Norfolk and Suffolk NHS Foundation Trust<br>Hiller, Rachel; University of Bath, Department of Psychology<br>Neil, Elsbeth; University of East Anglia, School of Social Work<br>Williams, Kevin; The Fostering Network<br>Rodgers, Luke; The Care Leaders<br>Wilson, Jon; Norfolk and Suffolk NHS Foundation Trust,<br>Wong, Geoff; Oxford University, Nuffield Department of Primary Care Health Sciences
Keywords:	Child & adolescent psychiatry < PSYCHIATRY, MENTAL HEALTH, PSYCHIATRY

SCHOLARONE™
Manuscripts

Improving the mental health and mental health support available to adolescents with social care-experience via low-intensity life story work: A realist review protocol

Simon P Hammond[ab]*, Claire Duddy[c], Ella Mickelburgh[b], Rachel Hillerd, Elsbeth Neil[e], Kevin Willaims[f], Luke Rodgers[g], Jon Wilson[b], Geoff Wong[c].

[a]School of Lifelong Learning, University of East Anglia (UEA), Norwich, United Kingdom (UK); [b]Norfolk and Suffolk NHS Foundation Trust, Norwich, UK, [c] Nuffield Department of Primary Care Health Sciences, University of Oxford, UK; [d]Department of Psychology, University of Bath, Bath, UK; [e]*Centre for Research on the Child and Family, School of Social Work, UEA, Norwich, UK; [f]The Fostering Network, London, UK; [g]The Care Leaders, Oxford, UK.*

**School of Education and Lifelong Learning, Faculty of Social Sciences, University of East Anglia. Norwich. NR4 7TJ. United Kingdom. Tel: (+44) (0)1603 591460. Email: S.Hammond@uea.ac.uk*

Keywords

Adolescent, life story work, realist review, mental health, mental health support

Word count: 3,697

For peer review only

Improving the mental health and mental health support available to adolescents with social care-experience via low-intensity life story work: A realist review protocol

Abstract

Introduction

Adolescents are the fastest growing age group entering social care and the group most at risk of mental ill-health. Life Story Work (LSW) is an existing transdiagnostic intervention used to improve the mental health of children and adolescents under the care of a local authority by assisting the processing of trauma. Yet LSW is poorly evidenced, lacks standardisation and focuses on younger children. LSW is also high-intensity, relying on specialist input over several months. Adolescent-focused low-intensity-LSW is a promising alternative. However, there is poor evidence on how LSW, let alone low-intensity-LSW should be delivered to adolescents. We aim to identify why, how, in what contexts, for whom and to what extent low-intensity-LSW interventions can be delivered to adolescents with care-experience.

Methods and analysis

We will undertake a realist review to: (1) develop an initial programme theory (PrT) of adolescent-focused low-intensity-LSW by consulting with two key expert panels (care-experienced and professional stakeholders), and by searching the literature to identify existing relevant theories; (2) undertake a comprehensive literature search to identify secondary data to develop and refine our emerging PrT; (3) select, extract and organise data; (4) synthesise evidence using a realist logic of analysis to develop the PrT, moving iteratively between the analysis of particular examples, refinement of the PrT and further iterative data searching, with further consultation with our expert panels (5) write up and share the refined PrT with our expert panels for their final comments. From this process will develop guidance to help improve the delivery of LSW to support the mental health needs of adolescents with care-experience.

Ethics and dissemination

Ethical approval is not required. Dissemination will include input from expert panels. We will develop academic, practice and youth focused outputs targeting adolescents, their carers, social, healthcare, and educational professionals, academics and policy-makers.

PROSPERO registration number: **CRD42021279816**

For peer review only - http://bmjopen.bmj.com/site/about/guidelines.xhtml

Strengths and limitations of this study

- This is the first realist review of adolescent-focused low-intensity Life Story Work and will improve our understanding of how this intervention may work in different settings and for different groups of adolescents with social care experience.
- In addition to including published research studies on Life Story Work interventions, this review will draw on learning from documents that describe local implementation and innovation in Life Story Work delivery.
- Our review will be improved by the contributions of our two separate Public Patient Involvement groups which feature young adults with care-experience and professionals as recipients and deliverers of Life Story Work.
- Our review may be limited by the richness and relevance of evidence available in the literature.
- The contribution of two contrasting PPI groups, with differing potential agendas, may create issues in consolidating our final programme theory.

For peer review only

Improving the mental health and mental health support available to adolescents with social care-experience via low-intensity life story work: A realist review protocol

Introduction and background

There are over 90,000 children and adolescents under the care of United Kingdom local authorities [1]. Adolescents are the fastest growing age group entering care in England [1] and the scale of their mental health needs is extraordinary for a 'non-clinical' population [2]. This group are up to six times more likely than their peers in the general population to experience mental ill-health [3] and 3-4 times more likely to attempt suicide [4]. Despite this, evidence indicates that the mental health needs of adolescents with social care-experience are underreported and undertreated [5].

The lifetime economic burden associated with outcomes stemming from child maltreatment, a central experience of many adolescents with care-experience, is estimated to be between £150-300 billion [6, 7]. This is more expensive than the combined economic burden of major medical illnesses [6]. Cost stems from the high lifetime use of social-care and health services and loss of productivity, including high rates of unemployment (e.g., almost 40% of adolescents who are not in education, training and employment [NEET] are care-experienced [6-8]). Finding ways to improve the mental health of adolescents with care-experience represents a clear health, social care and educational priority.

Interventions to improve the mental health of adolescents with care-experience do exist [9]. However, when framed by the hierarchy of evidence for therapeutic studies [10, 11], the majority are costly and viewed as having a 'low-quality' evidence-base [9, 12-14]. Being unable to answer vital questions such as what interventions work best, how, for whom, over what period and at what cost [12], makes the commissioning of services very difficult and increases crisis-based referrals [5, 13, 15].

A promising alternative to begin to address the unmet mental needs of adolescents with care-experience is the provision of quicker access to low-intensity services delivered at scale [4, 16-20]. Low-intensity interventions vary according to whether their delivery involves support from a healthcare professional (guided self-help) or not (non-facilitated self-help), as well as the mode (face-to-face and/or digital), duration and intensity of services provided [21, 22]. Early intervention and the delivery of low-intensity interventions by non-specialists could offer effective and cost-effective processes to improve mental health [23]. Evidence also indicates that early mental health interventions are more cost-effective than crisis-based referrals [24], reducing pressure on already stretched health and social care services and providing evidence-based approaches to the commissioners of services.

Hence, when seeking to develop and evaluate low-intensity mental health interventions for adolescents with care-experience to address what the National Institute for Health Care Excellence (NICE) describes as an "…urgent research priority…" [12], the first step is to understand how and why existing interventions 'work' or not in differing contexts, for whom and to what extent. This involves developing an

For peer review only - http://bmjopen.bmj.com/site/about/guidelines.xhtml

explicit Programme Theory (PrT), detailing the underlying assumptions about how an intervention is meant to work and what impacts are expected [25]. Developing this in-depth understanding is critical in the case of adolescents with care-experience as they are a heterogeneous group [9, 26]. Many will have complex histories and needs, meaning it is unlikely that an intervention with a single focus will address all of these needs [9]. This indicates that a transdiagnostic 'complex' intervention composed of several interacting components [27], capable of being delivered in a timely fashion and flexible enough to match the changing needs of the young person with care-experience may prove effective.

Life Story Work (LSW) is a complex, transdiagnostic intervention for improving the mental health of children and adolescents with care-experience. It is promoted in social care as a standard part of the care all children and adolescents with care-experience should receive. It is flexible, broad in focus and widely used, illustrated via legislation underpinning its usage [28-31]. LSW is grounded in assumptions that constructing a coherent narrative is important for processing trauma(s) and that integrating new or corrective information can reduce negative emotions related to trauma, transitions, and loss [32-42]. Typical LSW components include a therapeutic alliance (relationship with a trusted adult(s) capable of facilitating positive mental health), certain behaviours (individual or group therapeutic activities), procedures (prompts to action) and products (materials or artefacts).

However, despite the use of LSW being widely reported by people with care-experience, carers and professionals as valuable [43-53], relatively little is known about how it works and the extent to which it works, especially for adolescents with care-experience.

A 2006 systematic review by McKeown et al. on LSW in health and social care concluded that LSW had potentially far-reaching benefits but an "immature" evidence-base [47]. In a 2020 scoping review that examined the peer-reviewed empirical evidence for LSW, the authors concluded that despite LSW being a clear priority for all stakeholders, it lacked an accepted standard for delivery and robust implementation, and evidence of effectiveness and cost-effectiveness [54]. In reviewing the 17 included studies, the authors highlighted several weaknesses of the current evidence base [54]. These included assumptions of 'standard LSW' without clear standardisation protocols, conceptualisations of LSW that did not appreciate the longitudinal nature of care-experiences across the life course, age-related limitations in terms of how LSW was understood and a lack of opportunity for innovation in practice and delivery [54]. A more recent paper has also highlighted the need for a broader appreciation of the mechanisms through which delivery may occur [55].

A further weakness in the existing evidence base is that it does not sufficiently inform the development of LSW interventions. As noted by Hammond et al., the potential of low-intensity standardized transdiagnostic LSW approaches targeting adolescents is appealing, yet: "…without better evidence on what works best, how, for whom, over what period and at what cost we cannot move forward…" [54].

There is a clear need for research capable of building theoretically rich explanations of how low-intensity adolescent-focused-LSW works. Critically, this needs to be undertaken in a way that is flexible enough to recognise the varying home

For peer review only - http://bmjopen.bmj.com/site/about/guidelines.xhtml

circumstances in which adolescents experience social care. Theory-led research is important because it can deliver findings that are useable to service providers and transferable to the different settings and adolescents they work with [56].

Methods and analysis

The aim of the current research is to begin to address the unmet mental health needs of adolescents with care-experience by improving the evidence base for, and developing guidance to inform the delivery of adolescent-focused low-intensity-LSW interventions by asking:

"How, why, to what extent, for whom and in what circumstances can low-intensity LSW interventions, or elements of LSW interventions, be delivered to improve important and relevant outcomes for adolescents with care experience with mental health and wellbeing needs?"

This research question is operationalised into two main objectives:

1. Undertake a realist review, to develop and refine a realist PrT that explains how and why adolescent-focused low-intensity-LSW interventions (or elements of interventions) may or may not work for adolescents with care-experience and in what contexts.

2. Use the realist PrT to produce preliminary guidance on the nature of good practice when delivering adolescent-focused low-intensity-LSW to adolescents with care-experience and hence provide benefits for them, their carers and health, social care and educational professionals.

Realist review

We will address the research question and objectives by conducting a realist review. This approach will enable the team to deal with the complexity inherent in this research question, by accounting for the changing contexts of adolescents with care-experience across different settings and services. The realist approach is flexible enough to allow for the inclusion of the existing literature on LSW, alongside evidence that can provide transferable explanations for how and why other low-intensity mental health intervention strategies 'work' (and do not work) for adolescents with care-experience.

Patient and Public Involvement (PPI)

Patient and Public Involvement (PPI) has been central to the design of the study and will continue to be a central component of this review. The area of interest originated from lead author SH's time as a residential social care worker and the practice frustrations he faced when trying to engage adolescents with care-experience in LSW.

The project developed with PPI from discussions with team members with lived care leadership and lived care-experience LR and KW and adolescents with care-experience and practitioners from across England who highlighted numerous barriers to high-intensity LSW approaches and the "…childlike…" resources used with adolescents.

For peer review only - http://bmjopen.bmj.com/site/about/guidelines.xhtml

PPI co-applicants were integral to the inclusion of a young adults with care-experienced expert panel (known as the Care-experienced Content Expert Group (CECG)) alongside our multidisciplinary expert panel (known as the Content Expert Group (CEG)) which is comprised of professionals within the area. The CEG will meet three times during the project and provide insight into areas where the published and grey literature is lacking.

Our CCEG comprising of young adults with care-experience and CEG panel will meet as separate groups before being brought together to meet (face-to-face and/or remotely) and contribute to the research asynchronously after the meetings (via WhatsApp text and video messaging). The CCEG will review information and feed into the ongoing iteration of the PrT and lead on the youth-centred elements of its dissemination. In this way, we will ensure that any outputs are reflective of the requirements of relevant stakeholders, something unlikely to be achieved through the literature review alone.

Study design

We will follow a five-step process to conduct the review:

Step 1: Develop an initial PrT

We will develop an initial PrT, created through reading the documents we have found during exploratory searches undertaken whilst preparing this research project. We will develop the initial PrT through project team meetings, where we will discuss and debate what the initial PrT should be. We will then hold the first of our CCEG and CEG meetings, presenting our initial PrT for feedback and further refinement.

The purpose of this step is to locate any existing theories of why and how low-intensity LSW interventions work (or are thought to work), in what contexts they work, to what extent, and for whom. From these documents, we will identify any relevant existing theories of low-intensity LSW interventions and, where needed, will use techniques such as citation tracking and snowballing to obtain more data. At this stage we will also make use of project team knowledge and contacts to identify relevant additional sources of information [57]. These informal techniques are particularly useful since, as we have already established, we will need to search widely for theories of adolescent-focused low-intensity LSW interventions in the literature. Throughout this step, we will regularly discuss information gathered until the initial PrT of adolescent-focused low-intensity LSW is formed.

Step 2: Evidence search

Following the creation of our initial PrT, we will undertake a comprehensive search of the literature, seeking secondary data to develop and refine it. This will include a review of published and grey literature, including educational materials for professionals and/or carers produced by professional bodies.

For peer review only - http://bmjopen.bmj.com/site/about/guidelines.xhtml

We will design, pilot, and refine our search strategy with the input of an experienced information specialist CD. Our search strategy for this review will aim to update and build upon searches undertaken in June and July 2020 to inform a scoping review undertaken by members of our team [54]. We will run searches in multiple research databases including MEDLINE, PsycINFO, ASSIA, and Social Care Online along with relevant sources of grey literature. Our search strategy will include a comprehensive set of terms to describe the population of interest (adolescents, young people, looked-after, care-experience) and the intervention (LSW). In addition, we will undertake additional searches to identify documents containing data about other low-intensity mental health intervention strategies for adolescents with care-experience.

To maximise the inclusion of relevant material, we will employ complementary searching techniques as appropriate, including citation searching (snowballing) and searching for “sibling” or “kinship” papers associated with included documents [58, 59].

Additional searching may be undertaken in response to new information requirements identified, and until we have obtained sufficient data (‘theoretical saturation’) to conclude that our refined PrT is coherent and plausible.

Step 3: Selecting, extracting, and organising data

Documents will be selected using a three-step screening process. First, the lead reviewer will screen all potentially relevant documents retrieved by the search by title and abstract, against inclusion and exclusion criteria. Our initial inclusion criteria are outlined below in Table 1.

For peer review only - http://bmjopen.bmj.com/site/about/guidelines.xhtml

Table 1

Summary of eligibility criteria for realist review of adolescent-focused low-intensity Life Story Work for adolescents with care-experience

Inclusion criteria	*Exclusion criteria*
Population: Young people who are under the care of a local authority, young people who are 'looked after' or care experienced or adopted young people, or their parents/carers.<br><br>Intervention: LSW, including all activities involving recording, exploring, eliciting accounts of a care experienced person's life or personal history, to have an impact on their understanding of themselves and their identify<br><br>And/or<br><br>Low-intensity interventions that aim to address a mental health or wellbeing need<br><br>Document type/study design: any<br><br>Other: English language only	Research focused solely on parenting style, communicative openness in foster or adoptive families, contact with birth family members

Second, the full text of documents that met the inclusion criteria in the initial screen will be obtained and screened against the inclusion and exclusion criteria. Third, those that fulfil inclusion criteria will be read in detail and our final decision on inclusion in the review will be based on the criteria of *relevance* (does a document contain data that can contribute to the development of the PrT?) and *rigour* (were the methods used to generate the data trustworthy and credible?)[60]. To ensure consistency in the application of the inclusion criteria we will use a process we have used before [61] and a 10% random sample of documents will be screened in duplicate at each stage by another member of the project team. Any discrepancies will be resolved through wider project team discussions.

Where necessary we will use established quality appraisal tools to judge the rigour of the data in included documents. For example, we will do this when a document contributes a substantial amount of data to our PrT and hence it is important for us to be able to trust these data by assessing the rigour of the methods used to generate the data. Where there is uncertainty as to how to judge rigour, we will predominantly consider the relevance of the data. In other words, we will likely include any relevant

For peer review only - http://bmjopen.bmj.com/site/about/guidelines.xhtml

data. We will take this approach as even data that is of questionable quality may still provide relevant information to inform PrT development. To ensure that our PrT continues to provide plausible explanations for adolescent-focused low-intensity LSW, we will use an additional process for 'quality control'. That is, we will judge the explanatory plausibility of the PrT using the criteria of consilience, simplicity and analogy [62]. Despite these measures, threats to the plausibility of our PrT may still occur, particularly if sections of it are based predominantly on data that we would judge (globally) to be of questionable 'rigour'. In such cases, we will highlight and report as a limitation.

Data from all relevant full text documents will be extracted using a suitably designed and piloted standardised data collection process. We anticipate key characteristics of each included document will be extracted into an Excel spreadsheet, and that the full text of documents will be uploaded to NVivo (a qualitative data analysis software) so relevant data can be organised and coded. Coding will involve extracting relevant sections of text from included documents according to how this data can contribute to PrT development.

Step 4: Synthesising evidence
Data analysis will involve the use of a realist logic of analysis with the goal of using the data from the documents to further develop the initial PrT developed in Step 1. Data coding will be deductive (informed by the initial PrT), inductive (coming from the data within included documents) and retroductive (where inferences are made about underlying causal processes or mechanisms). Drawing on previous work [63], we will use a series of questions about the relevance and rigour of content within documents as part of our process of analysis, as set out below:

Relevance:

- Are sections of text within this document relevant to PrT development?

Rigour (judgements about trustworthiness):

- Are these data sufficiently trustworthy to warrant making changes to any aspect of the initial and emerging PrT?

Interpretation of meaning:

- If the section of text is relevant and trustworthy enough, do its contents provide data that may be interpreted as functioning as context, mechanism or outcome?

Interpretations and judgements about Context-Mechanism-Outcome-Configurations:

- For the data that has been interpreted as functioning as context, mechanism or outcome, which Context-Mechanism-Outcome-Configuration (CMOC) (partial or complete) does it belong to?
- Are there further data to inform this particular CMOC contained within this document or other documents? If so, which other documents?
- How does this particular CMOC relate to other CMOCs that have already been developed?

For peer review only - http://bmjopen.bmj.com/site/about/guidelines.xhtml

Interpretations and judgements about PrT:

- How does this (full or partial) CMOC relate to the PrT?
- Within this same document are there data which informs how the CMOC relates to the PrT? If not, are there data in other documents? Which ones?
- Considering this particular CMOC and any supporting data, does the PrT need to be changed?

Data to inform our interpretation of the relationships between contexts, mechanisms and outcomes will be sought not just within the same source, but across sources (e.g., mechanisms inferred from one document could help to explain the way contexts influenced outcomes for an intervention in another). Synthesising data from different documents is often necessary to compile CMOCs, since not all parts of the configurations can be found in the same document.

During the review, we will move iteratively between the analysis of particular examples, refinement of the PrT, and further iterative data searching to test particular theories (where needed).

During this step, we will hold our second CCEG and CEG meetings to discuss the literature, and sense check the developing PrT. The PrT and a summary of the literature will be discussed with these groups who will be asked to comment on its resonance with their perspectives and its implications for preliminary guidance. Wherever possible, we will address any gaps in the theory that persist (e.g., through additional literature searches).

Step 5: Finalising the PrT and drawing conclusions

Near the end of the review, the refined PrT will be written up and shared during the final CCEG and CEG meetings for final comments. We will seek input to ensure the outputs we produce (outlined in the section below) are useful to all stakeholders and disseminated across 'lay', professional and academic networks.

Ethics and dissemination

Ethical approval is not required as the realist review is secondary research.

The main outputs of this research will be an evidence-based PrT of adolescent-focused low-intensity-LSW that will inform our preliminary guidance which can be used to optimise any pre-existing practice immediately. We will share our final PrT using text, summary tables, a logic model and where appropriate youth focused information clips and/or infographics to summarise individual papers/reports and draw insights across papers/reports.

For academic, clinical and social care audiences, we will produce peer-reviewed journal articles, including those detailing the process and findings of the realist review and establishing the requirements for effective adolescent-focused low-intensity LSW. For other professional audiences we will actively share our preliminary guidance on

For peer review only - http://bmjopen.bmj.com/site/about/guidelines.xhtml

the nature of good practice when delivering adolescent-focused low-intensity-LSW. This will take the form of articles and blogs.

For peer review only

For peer review only - http://bmjopen.bmj.com/site/about/guidelines.xhtml

References

1. Department for Education, *Children looked after in England including adoption, year ending 31 March 2019.*, D.f. Education, Editor. 2019: London, UK.
2. Tarren-Sweeney, M., *The mental health of children in out-of-home care.* Current Opinion in Psychiatry, 2008. **21**(4): p. 345-349.
3. Meltzer, H., et al., *The Mental Health of Young People Looked After by Local Authorities in England.* , T.T.S. Office), Editor. 2003: Londin.
4. Evans, R., et al., *Comparison of suicidal ideation, suicide attempt and suicide in children and young people in care and non-care populations: Systematic review and meta-analysis of prevalence.* Children and Youth Services Review, 2017. **82**: p. 122-129.
5. CQC, *The state of mental health services 2014 - 2017: Findings from CQCs programme of comprehensive inspections of specialist mental health services.* 2018.
6. Fang, X., et al., *The economic burden of child maltreatment in the United States and implications for prevention.* Child abuse & neglect, 2012. **36**(2): p. 156-165.
7. Conti, G., et al., *The economic cost of child maltreatment in the UK : a preliminary study*. 2017, NSPCC: London.
8. Education, D.f., *Charactersitics of young poeple who are long-term NEET*. 2018: London.
9. Luke, N., et al., *What works in preventing and treating poor mental health in looked after children?* 2014, Rees Centre: London, UK.
10. Evans, D., *Hierarchy of evidence: a framework for ranking evidence evaluating healthcare interventions.* Journal of Clinical Nursing, 2003. **12**(1): p. 77-84.
11. CEBM. *Oxford Centre for Evidence-based Medicine - Levels of Evidence (March 2009)*. 2009 [cited 2020 02.03.2020]; Available from: https://www.cebm.net/2009/06/oxford-centre-evidence-based-medicine-levels-evidence-march-2009/.
12. NICE. *Child abuse and neglect: NICE guideline [NG76]*. 2017 [cited 2020 10.02.2020]; Available from: https://www.nice.org.uk/guidance/ng76.
13. Department of Health and Department for Education, *Transforming children and young people's mental health provision: Green paper*. 2017.
14. The Education Committee, *Mental health and well-being of looked-after children*, H.o.C.E. Committee, Editor. 2016, House of Commons: London, UK.
15. Department of Health, *A Framework for mental health research*, D.o. Health, Editor. 2017: London, UK.
16. NHS. *Mental Health of Children and Young People in England, 2017*. 2018 [cited 2020 23.02.2020]; Available from: https://digital.nhs.uk/data-and-information/publications/statistical/mental-health-of-children-and-young-people-in-england/2017/2017#key-facts.
17. Action for Children, National Children's Bureau, and The Children's Society, *Turning the tide: Reversing the move to late intervention spending in children and young people's services*. 2017: London, UK.
18. Fairburn, C.G. and V. Patel, *The impact of digital technology on psychological treatments and their dissemination.* Behaviour research and therapy, 2017. **88**: p. 19-25.
19. Hollis, C., et al., *Identifying research priorities for digital technology in mental health care: results of the James Lind Alliance Priority Setting Partnership.* The Lancet Psychiatry, 2018. **5**(10): p. 845-854.
20. James, K., *Remote mental health interventions for young people: a rapid review of the evidence*. 2020, Youth Access: Championing advise and counselling: London.
21. NICE. *Common mental health problems: identification and pathways to care: Clinical guideline [CG123]* 2011 [cited 2020 22.02.2020]; Available from: https://www.nice.org.uk/guidance/cg123/chapter/Appendix-E-Glossary.
22. Kazdin, A.E. and S.M. Rabbitt, *Novel Models for Delivering Mental Health Services and Reducing the Burdens of Mental Illness.* Clinical Psychological Science, 2013. **1**(2): p. 170-191.

For peer review only - http://bmjopen.bmj.com/site/about/guidelines.xhtml

23. NHS, *The Improving Access to Psychological Therapies Manual*. 2019, National Collagorating Centre for Mental Health: London, UK.
24. Bellis, M.A., et al., *Life course health consequences and associated annual costs of adverse childhood experiences across Europe and North America: a systematic review and meta-analysis.* The Lancet Public Health, 2019. **4**(10): p. e517-e528.
25. Pawson, R., et al., *Realist review--a new method of systematic review designed for complex policy interventions.* J Health Serv Res Policy, 2005. **10 Suppl 1**: p. 21-34.
26. Sinclair I, et al., *The pursuit of permanence: A study of the English care system*. 2007, London, UK: Jessica Kingsley Publishers.
27. Craig, P., et al., *Developing and evaluating complex interventions: the new Medical Research Council guidance.* BMJ (Clinical research ed.), 2008. **337**: p. a1655-a1655.
28. Adoption and Children Act. *Adoption and Children Act 2002*. 2002 [cited 2020 02.03.2020]; Available from: http://www.legislation.gov.uk/ukpga/2002/38/contents.
29. Children Act. *Children Act 1989*. 1989 [cited 2020 02.03.2020]; Available from: http://www.legislation.gov.uk/ukpga/1989/41/contents.
30. Department for Education, *Guide to the Children's Homes Regulations including the quailty standards*, D.f. Education, Editor. 2015: London, UK.
31. The Fostering Services (England) Regulations. *The Fostering Services (England) Regulations 2011*. 2011 [cited 2020 02.03.2020]; Available from: http://www.legislation.gov.uk/uksi/2011/581/contents/made.
32. Ehlers, A. and D.M. Clark, *A cognitive model of posttraumatic stress disorder.* Behav Res Ther, 2000. **38**(4): p. 319-45.
33. Salmond, C.H., et al., *The nature of trauma memories in acute stress disorder in children and adolescents.* Journal of Child Psychology and Psychiatry, 2011. **52**(5): p. 560-570.
34. van Os, E.C.C., et al., *Finding Keys: A Systematic Review of Barriers and Facilitators for Refugee Children's Disclosure of Their Life Stories.* Trauma, Violence, & Abuse, 2020. **21**(2): p. 242-260.
35. Fivush, R., *Children's recollections of traumatic and nontraumatic events.* Dev Psychopathol, 1998. **10**(4): p. 699-716.
36. Mundorf, E.S. and S.C. Paivio, *Narrative quality and disturbance pre- and post-emotion-focused therapy for child abuse trauma.* J Trauma Stress, 2011. **24**(6): p. 643-50.
37. Tuval-Mashiach, R., et al., *Coping with Trauma: Narrative and Cognitive Perspectives.* Psychiatry: Interpersonal and Biological Processes, 2004. **67**(3): p. 280-293.
38. Cohen, J.A. and A.P. Mannarino, *Trauma-Focused Cognitive Behavioural Therapy for Children and Parents.* Child and Adolescent Mental Health, 2008. **13**(4): p. 158-162.
39. Foa, E.B. and M.J. Kozak, *Emotional processing of fear: Exposure to corrective information.* Psychological Bulletin, 1986. **99**(1): p. 20-35.
40. Foa, E.B. and C.P. McLean, *The Efficacy of Exposure Therapy for Anxiety-Related Disorders and Its Underlying Mechanisms: The Case of OCD and PTSD.* Annu Rev Clin Psychol, 2016. **12**: p. 1-28.
41. Rose, R., *Life Story Therapy With Traumatized Children: A Model for Practice.* . 2012, London, UK: Jessica Kingsley Publishers
42. Askelund, A.D., et al., *Positive memory specificity is associated with reduced vulnerability to depression.* Nat Hum Behav, 2019. **3**(3): p. 265-273.
43. Baynes, P., *Untold Stories: A Discussion of Life Story Work.* Adoption & Fostering, 2008. **32**(2): p. 43-49.
44. Buchanan, A., *The experience of Life Story Work: Reflections of Young People Leaving Care*. 2014, Cardiff University: Cardiff, UK. p. 181.
45. Cook-Cottone, C. and M. Beck, *A Model for Life-Story Work: Facilitating the Construction of Personal Narrative for Foster Children.* Child and Adolescent Mental Health, 2007. **12**(4): p. 193-195.

For peer review only - http://bmjopen.bmj.com/site/about/guidelines.xhtml

46. Hooley, K., L. Stokes, and H. Combes, *Life story work with looked after and adopted children: how professional training and experience determine perceptions of its value.* Adoption & Fostering, 2016. **40**(3): p. 219-233.
47. McKeown, J., A. Clarke, and J. Repper, *Life story work in health and social care: systematic literature review.* Journal of Advanced Nursing, 2006. **55**(2): p. 237-247.
48. NSPCC. *How Life Story Work works*. 2020 [cited 2020 10.02.2020]; Available from: https://learning.nspcc.org.uk/services-children-families/life-story-work/#article-top.
49. Ryan, T. and R. Walker, *Life Story Work*. 2016, London: CoramBAAF.
50. Willis, R. and S. Holland, *Life Story Work: Reflections on the Experience by Looked after Young People.* Adoption & Fostering, 2009. **33**(4): p. 44-52.
51. Wrench, K. and L. Naylor, *Life Story Work with Children Who are Fostered or Adopted: Creative Ideas and Activities*. 2013, London: Jessica Kingsley.
52. Watson, D., S. Latter, and R. Bellew, *Adopters' views on their children's life story books.* Adoption & Fostering, 2015. **39**(2): p. 119-134.
53. Watson, D.L., S. Latter, and R. Bellew, *Adopted children and young people's views on their life storybooks: The role of narrative in the formation of identities.* Children and Youth Services Review, 2015. **58**: p. 90-98.
54. Hammond, S.P., J. Young, and C. Duddy, *Life story work for children and young people with care experience: A scoping review.* Developmental Child Welfare, 2020. **2**(4): p. 293-315.
55. Hammond, S.P., N.J. Cooper, and P. Jordan, *Mental health, identity and informal education opportunities for adolescents with experience of living in state care: a role for digital storytelling.* Cambridge Journal of Education, 2021: p. 1-20.
56. Wong, G., et al., *Realist methods in medical education research: what are they and what can they contribute?* Med Educ, 2012. **46**(1): p. 89-96.
57. Greenhalgh, T. and R. Peacock, *Effectiveness and efficiency of search methods in systematic reviews of complex evidence: audit of primary sources.* BMJ, 2005. **331**(7524): p. 1064-1065.
58. Booth, A., et al., *Towards a methodology for cluster searching to provide conceptual and contextual "richness" for systematic reviews of complex interventions: case study (CLUSTER).* BMC Medical Research Methodology, 2013. **13**(1): p. 118.
59. Booth, A., J. Wright, and B. S, *Scoping and searching to support realist approaches.* , in *Doing Realist Research.* , Emmel N, et al., Editors. 2018, SAGE Publications Ltd: London, UK. p. 148-65.
60. Pawson, R., *Evidence-Based Policy: A realist perspective*. 2006, London, UK: Sage.
61. Nyssen, O.P., et al., *Does therapeutic writing help people with long-term conditions? Systematic review, realist synthesis and economic considerations.* Health Technol Assess, 2016. **20**(27): p. vii-xxxvii, 1-367.
62. Wong, G., *Looking for needles in haystacks*, in *Doing Realist Research*, N. Emmel, et al., Editors. 2018, SAGE: London.
63. Papoutsi, C., et al., *Health Services and Delivery Research*, in *Interventions to improve antimicrobial prescribing of doctors in training (IMPACT): a realist review*. 2018, NIHR Journals Library

For peer review only

For peer review only - http://bmjopen.bmj.com/site/about/guidelines.xhtml

Author's contributions

Contributors SH conceived the study with input from GW, RH, LR, KW, EN, CD and JW. SH and EM and wrote the first draft of this protocol, and GW, CD, RH, EN, KW, JW and LR provided criticism and refinement. CD designed the search strategy. All the authors have read and approved the final manuscript.

Funding

This project is funded by the National Institute for Health Research (NIHR) Research for Patient Benefit Programme (Grant Reference Number NIHR 201963).

Disclaimer

The views expressed in this publication are those of the author(s) and not necessarily those of the NIHR or the Department of Health and Social Care.

Competing interests

SH offers consultancy services in social media and Digital Life Story Work via www.digitallifestorywork.co.uk. LR is Director of Strategy of The Care Leaders, KW is the CEO of The Fostering Network. The remaining authors declare that they have no known competing financial interests or personal relationships that could have appeared to influence the work reported in this paper.

For peer review only

BMJ Open

Improving the mental health and mental health support available to adolescents with social care-experience via low-intensity life story work: A realist review protocol

Journal:	*BMJ Open*
Manuscript ID	bmjopen-2021-058424.R1
Article Type:	Protocol
Date Submitted by the Author:	11-Feb-2022
Complete List of Authors:	Hammond, Simon; University of East Anglia, School of Education and Lifelong Learning<br>Duddy, Claire; University of Oxford, Nuffield Department of Primary Care Health Sciences<br>Mickleburgh, Ella; Norfolk and Suffolk NHS Foundation Trust<br>Hiller, Rachel; University College London, Division of Psychology and Language Sciences<br>Neil, Elsbeth; University of East Anglia, School of Social Work<br>Williams, Kevin; The Fostering Network<br>Rodgers, Luke; The Care Leaders<br>Wilson, Jon; Norfolk and Suffolk NHS Foundation Trust,<br>Wong, Geoff; Oxford University, Nuffield Department of Primary Care Health Sciences
**Primary Subject Heading**:	Mental health
Secondary Subject Heading:	Mental health
Keywords:	Child & adolescent psychiatry < PSYCHIATRY, MENTAL HEALTH, PSYCHIATRY

SCHOLARONE™
Manuscripts

Improving the mental health and mental health support available to adolescents with social care-experience via low-intensity life story work: A realist review protocol

Simon P Hammond[ab*], Claire Duddy[c], Ella Mickleburgh[b], Rachel Hiller[de], Elsbeth Neil[f], Kevin Williams[g], Luke Rodgers[h], Jon Wilson[b], Geoff Wong[c].

[a]*School of Lifelong Learning, University of East Anglia (UEA), Norwich, United Kingdom (UK);* [b]*Norfolk and Suffolk NHS Foundation Trust, Norwich, UK,* [c]*Nuffield Department of Primary Care Health Sciences, University of Oxford, UK;* [d]Division of Psychology and Language Sciences, University College London, UK; [e]Anna Freud National Centre for Children and Families, London; [f]*Centre for Research on the Child and Family, School of Social Work, UEA, Norwich, UK;* [g]*The Fostering Network, London, UK;* [h]*The Care Leaders, Oxford, UK.*

**School of Education and Lifelong Learning, Faculty of Social Sciences, University of East Anglia. Norwich. NR4 7TJ. United Kingdom. Tel: (+44) (0)1603 591460. Email: S.Hammond@uea.ac.uk*

Keywords

Adolescent, life story work, realist review, mental health, mental health support

Word count: 3,899

For peer review only - http://bmjopen.bmj.com/site/about/guidelines.xhtml

Improving the mental health and mental health support available to adolescents with social care-experience via low-intensity life story work: A realist review protocol

Abstract

Introduction

Adolescents are the fastest growing group entering social care and are most at risk of mental ill-health. Life Story Work (LSW) is an existing transdiagnostic intervention thought to improve the wellbeing and mental health of children and adolescents under the care of a local authority by assisting the processing of trauma. Yet LSW is poorly evidenced, lacks standardisation and focuses on younger children. LSW is also high-intensity, relying on specialist input over several months. Adolescent-focused low-intensity-LSW is a promising alternative. However, there is poor evidence on how LSW, let alone low-intensity-LSW should be delivered to adolescents. We aim to identify why, how, in what contexts, for whom and to what extent low-intensity-LSW interventions can be delivered to adolescents with care-experience.

Methods and analysis

Undertaking a realist review, we will: (1) develop an initial programme theory (PrT) of adolescent-focused low-intensity-LSW by consulting with two key expert panels (care-experienced and professional stakeholders), and by searching the literature to identify existing relevant theories; (2) undertake a comprehensive literature search to identify secondary data to develop and refine our emerging PrT. Searches will be run between 12/2021-06/2022 in databases including MEDLINE, PsycINFO, ASSIA and relevant sources of grey literature; (3) select, extract and organise data; (4) synthesise evidence using a realist logic of analysis and undertake further iterative data searching and consultation with our expert panels; (5) write up and share the refined PrT with our expert panels for their final comments. From this process guidance will be developed to help improve the delivery of LSW to support the mental health needs of adolescents with care-experience.

Ethics and dissemination

Ethical approval is not required. Dissemination will include input from expert panels. We will develop academic, practice and youth focused outputs targeting adolescents, their carers, social, healthcare, and educational professionals, academics, and policymakers.

PROSPERO registration number: **CRD42021279816**

For peer review only - http://bmjopen.bmj.com/site/about/guidelines.xhtml

Strengths and limitations of this study

- This is the first realist review of adolescent-focused low-intensity Life Story Work and will improve our understanding of how this intervention may work in different settings and for different groups of adolescents with social care experience.
- Our review includes contributions from two separate Public Patient Involvement groups featuring young adults with care-experience and professionals as recipients and deliverers of Life Story Work.
- The contribution of two contrasting PPI groups, with differing potential agendas, may create issues in consolidating our final programme theory.
- Our review may be limited by the richness and relevance of evidence available in the literature.

For peer review only

Improving the mental health and mental health support available to adolescents with social care-experience via low-intensity life story work: A realist review protocol

Introduction and background

There are over 90,000 children and adolescents under the care of United Kingdom (UK) local authorities [1]. Adolescents are the fastest growing age group entering care in England [1] and the scale of their mental health needs is extraordinary for a 'non-clinical' population [2]. This group are up to six times more likely than their peers in the general population to experience mental ill-health [3] and 3-4 times more likely to attempt suicide [4]. Despite this, evidence indicates that the mental health needs of adolescents with social care-experience are underreported and undertreated [5].

The lifetime economic burden associated with outcomes stemming from child maltreatment, a central experience of many adolescents with care-experience, is estimated to be between £150-300 billion [6, 7]. This is more expensive than the combined economic burden of major medical illnesses [6]. Cost stems from the high lifetime use of social-care and health services and loss of productivity, including high rates of unemployment (e.g., almost 40% of adolescents who are not in education, training and employment [NEET] are care-experienced [6-8]). Finding ways to improve the mental health of adolescents with care-experience represents a clear health, social care and educational priority.

Interventions to improve the mental health of adolescents with care-experience do exist [9]. However, when framed by the hierarchy of evidence for therapeutic studies [10, 11], the majority are costly and viewed as having a 'low-quality' evidence-base [9, 12-14]. Being unable to answer vital questions such as what interventions work best, how, for whom, over what period and at what cost [12], makes the commissioning of services very difficult and increases crisis-based referrals [5, 13, 15].

A promising alternative to begin to address the unmet mental needs of adolescents with care-experience is the provision of quicker access to low-intensity services delivered at scale [4, 16-20]. Low-intensity interventions vary according to whether their delivery involves support from a healthcare professional (guided self-help) or not (non-facilitated self-help), as well as the mode (face-to-face and/or digital), duration and intensity of services provided [21, 22]. Early intervention and the delivery of low-intensity interventions by non-specialists could offer effective and cost-effective processes to improve mental health [23]. Evidence also indicates that early mental health interventions are more cost-effective than crisis-based referrals [24], reducing pressure on already stretched health and social care services and providing evidence-based approaches to the commissioners of services.

Hence, when seeking to develop and evaluate low-intensity mental health interventions for adolescents with care-experience to address what the National Institute for Health Care Excellence (NICE) describes as an "…urgent research priority…" [12], the first step is to understand how and why existing interventions 'work' or not in differing contexts, for whom and to what extent. This involves developing an

For peer review only - http://bmjopen.bmj.com/site/about/guidelines.xhtml

explicit Programme Theory (PrT), detailing the underlying assumptions about how an intervention is meant to work and what impacts are expected [25]. Developing this in-depth understanding is critical in the case of adolescents with care-experience as they are a heterogeneous group [9, 26]. Many will have complex histories and needs, meaning it is unlikely that an intervention with a single focus will address all of these needs [9]. This indicates that a transdiagnostic 'complex' intervention composed of several interacting components [27], capable of being delivered in a timely fashion and flexible enough to match the changing needs of the young person with care-experience may prove effective.

Life Story Work (LSW) is an existing transdiagnostic intervention thought to improve the wellbeing and mental health of children and adolescents under the care of a local authority by assisting the processing of trauma. It is promoted in social care as a standard part of the care all children and adolescents with care-experience should receive. It is flexible, broad in focus and widely used, illustrated via legislation underpinning its usage [28-31]. LSW is grounded in assumptions that constructing a coherent narrative is important for processing trauma(s) and that integrating new or corrective information can reduce negative emotions related to trauma, transitions, and loss [32-42]. Typical LSW components include a therapeutic alliance (relationship with a trusted adult(s) capable of facilitating positive mental health), certain behaviours (individual or group therapeutic activities), procedures (prompts to action) and products (materials or artefacts).

However, despite the use of LSW being widely reported by people with care-experience, carers, and professionals as valuable [43-53], relatively little is known about how it works and the extent to which it works, especially for adolescents with care-experience.

A 2006 systematic review by McKeown et al. on LSW in health and social care concluded that LSW had potentially far-reaching benefits but an "immature" evidence-base [47]. In a 2020 scoping review that examined the peer-reviewed empirical evidence for LSW, the authors concluded that despite LSW being a clear priority for all stakeholders, it lacked an accepted standard for delivery and robust implementation, and evidence of effectiveness and cost-effectiveness [54]. In reviewing the 17 included studies, the authors highlighted several weaknesses of the current evidence base [54]. These included assumptions of 'standard LSW' without clear standardisation protocols, conceptualisations of LSW that did not appreciate the longitudinal nature of care-experiences across the life course, age-related limitations in terms of how LSW was understood and a lack of opportunity for innovation in practice and delivery [54]. A more recent paper has also highlighted the need for a broader appreciation of the mechanisms through which delivery may occur [55].

A further weakness in the existing evidence base is that it does not sufficiently inform the development of LSW interventions. As noted by Hammond et al., the potential of low-intensity standardized transdiagnostic LSW approaches targeting adolescents is appealing, yet: "…without better evidence on what works best, how, for whom, over what period and at what cost we cannot move forward…" [54].

In summary, adolescents are the quickest growing age group entering UK social care [1]. Adolescents with care-experience are up to six times more likely than their peers

For peer review only - http://bmjopen.bmj.com/site/about/guidelines.xhtml

in the general population to experience mental ill-health, with their mental health needs often remaining unmet with significant individual, societal and economic life-long consequences [3-8]. LSW is a widely accepted and currently used intervention which is assumed to promote mental health, but its' evidence-base is limited [9, 46, 47, 54, 56]. Conventional LSW interventions are costly [43, 45, 48, 52], tend to focus on younger children with care-experience [54-58] and, like other interventions in children's social care, lack focus on the longer-term impact and attributable outcomes [59].

Adolescent-focused low-intensity LSW interventions have the potential to improve the mental health of adolescents with care-experience [55]. However, there is a clear need for research capable of building theoretically rich explanations of how low-intensity adolescent-focused-LSW works. Critically, this needs to be undertaken in a way that is flexible enough to recognise the varying home circumstances in which adolescents experience social care. Theory-led research is important because it can deliver findings that are useable to service providers and transferable to the different settings and adolescents they work with [60].

Methods and analysis

The aim of the current research is to begin to address the unmet mental health needs of adolescents with care-experience by improving the evidence base for, and developing guidance to inform the delivery of adolescent-focused low-intensity-LSW interventions. We know there is robust evidence that constructing coherent narratives are important mechanisms for processing trauma memories. However, the specific use of this method (LSW) with this population (adolescents with care-experience) in these settings (social care) is poorly informed theoretically and empirically. We will begin to address these gaps by asking:

"How, why, to what extent, for whom and in what circumstances can low-intensity LSW interventions, or elements of LSW interventions, be delivered to improve important and relevant outcomes for adolescents with care experience with mental health and wellbeing needs?"

This research question is operationalised into two main objectives:

1. Undertake a realist review, to develop and refine a realist PrT that explains how and why adolescent-focused low-intensity-LSW interventions (or elements of interventions) may or may not work for adolescents with care-experience and in what contexts.

2. Use the realist PrT to produce preliminary guidance on the nature of good practice when delivering adolescent-focused low-intensity-LSW to adolescents with care-experience and hence provide benefits for them, their carers and health, social care and educational professionals.

Realist review

We will address the research question and objectives by conducting a realist review. This approach will enable the team to deal with the complexity inherent in this research question, by accounting for the changing contexts of adolescents with care-experience

For peer review only - http://bmjopen.bmj.com/site/about/guidelines.xhtml

across different settings and services. The realist approach is flexible enough to allow for the inclusion of the existing literature on LSW, alongside evidence that can provide transferable explanations for how and why other low-intensity mental health intervention strategies 'work' (and do not work) for adolescents with care-experience. We will follow the current RAMESES quality and publication standards for realist reviews in this project [61].

Patient and Public Involvement (PPI)

Patient and Public Involvement (PPI) has been central to the design of the study and will continue to be a central component of this review. The area of interest originated from lead author SH's time as a residential social care worker and the practice frustrations he faced when trying to engage adolescents with care-experience in LSW.

The project developed with PPI from discussions with team members with lived care leadership and lived care-experience LR and KW and adolescents with care-experience and practitioners from across England who highlighted numerous barriers to high-intensity LSW approaches and the "…childlike…" resources used with adolescents.

PPI co-applicants were integral to the inclusion of a young adults with care-experienced expert panel (known as the Care-experienced Content Expert Group (CCEG)) alongside our multidisciplinary expert panel (known as the Content Expert Group (CEG)) which is comprised of professionals within the area. The CCEG and CEG will meet three times during the project and provide insight into areas where the published and grey literature is lacking.

Our CCEG comprising of young adults with care-experience and CEG panel will meet as separate groups before being brought together to meet (face-to-face and/or remotely) and contribute to the research asynchronously after the meetings (via WhatsApp text and video messaging). The CCEG will review information and feed into the ongoing iteration of the PrT and lead on the youth-centred elements of its dissemination. In this way, we will ensure that any outputs are reflective of the requirements of relevant stakeholders, something unlikely to be achieved through the literature review alone.

Study design

We will follow a five-step process to conduct the review:

Step 1: Develop an initial PrT

We will develop an initial PrT, created through reading the documents we have found during exploratory searches undertaken whilst preparing this research project. We will develop the initial PrT through project team meetings, where we will discuss and debate what the initial PrT should be. We will then hold the first of our CCEG and CEG meetings, presenting our initial PrT for feedback and further refinement.

For peer review only - http://bmjopen.bmj.com/site/about/guidelines.xhtml

The purpose of this step is to locate any existing theories of why and how low-intensity LSW interventions work (or are thought to work), in what contexts they work, to what extent, and for whom. From these documents, we will identify any relevant existing theories of low-intensity LSW interventions and, where needed, will use techniques such as citation tracking and snowballing to obtain more data. At this stage we will also make use of project team knowledge and contacts to identify relevant additional sources of information [62]. These informal techniques are particularly useful since, as we have already established, we will need to search widely for theories of adolescent-focused low-intensity LSW interventions in the literature. Throughout this step, we will regularly discuss information gathered until the initial PrT of adolescent-focused low-intensity LSW is formed.

Step 2: Evidence search

Following the creation of our initial PrT, we will undertake a comprehensive search of the literature, seeking secondary data to develop and refine it. This will include a review of published and grey literature, including educational materials for professionals and/or carers produced by professional bodies.

We will design, pilot, and refine our search strategy with the input of an experienced information specialist CD. Our search strategy for this review will aim to update and build upon searches undertaken in June and July 2020 to inform a scoping review undertaken by members of our team [54]. We will run searches in multiple research databases including MEDLINE, PsycINFO, ASSIA, and Social Care Online along with relevant sources of grey literature. Our search strategy will include a comprehensive set of terms to describe the population of interest (adolescents, young people, looked-after, care-experience) and the intervention (LSW). In addition, we will undertake additional searches to identify documents containing data about other low-intensity mental health intervention strategies for adolescents with care-experience. For full details of the search strategies for these searches please see supplemental file 1.

To maximise the inclusion of relevant material, we will employ complementary searching techniques as appropriate, including citation searching (snowballing) and searching for “sibling” or “kinship” papers associated with included documents [63, 64].

Additional searching may be undertaken in response to new information requirements identified, and until we have obtained sufficient data (‘theoretical saturation’) to conclude that our refined PrT is coherent and plausible.

Step 3: Selecting, extracting, and organising data

Documents will be selected using a three-step screening process. First, the lead reviewer will screen all potentially relevant documents retrieved by the search by title and abstract, against inclusion and exclusion criteria. Our initial inclusion criteria are outlined below in Table 1.

For peer review only - http://bmjopen.bmj.com/site/about/guidelines.xhtml

Table 1

Summary of eligibility criteria for realist review of adolescent-focused low-intensity Life Story Work for adolescents with care-experience

Inclusion criteria	*Exclusion criteria*
Population: Young people who are under the care of a local authority, young people who are 'looked after' or care experienced or adopted young people, or their parents/carers.<br><br>Intervention: LSW, including all activities involving recording, exploring, eliciting accounts of a care experienced person's life or personal history, to have an impact on their understanding of themselves and their identify<br><br>And/or<br><br>Low-intensity interventions that aim to address a mental health or wellbeing need<br><br>Document type/study design: any<br><br>Other: English language only	Research focused solely on parenting style, communicative openness in foster or adoptive families, contact with birth family members

Second, the full text of documents that met the inclusion criteria in the initial screen will be obtained and screened against the inclusion and exclusion criteria. Third, those that fulfil inclusion criteria will be read in detail and our final decision on inclusion in the review will be based on the criteria of *relevance* (does a document contain data that can contribute to the development of the PrT?) and *rigour* (were the methods used to generate the data trustworthy and credible?)[65]. To ensure consistency in the application of the inclusion criteria we will use a process we have used before [66] and a 10% random sample of documents will be screened in duplicate at each stage by another member of the project team. Any discrepancies will be resolved through wider project team discussions.

Where necessary we will use established quality appraisal tools to judge the rigour of the data in included documents. For example, we will do this when a document contributes a substantial amount of data to our PrT and hence it is important for us to be able to trust these data by assessing the rigour of the methods used to generate the data. Where there is uncertainty as to how to judge rigour, we will predominantly consider the relevance of the data. In other words, we will likely include any relevant

For peer review only - http://bmjopen.bmj.com/site/about/guidelines.xhtml

data. We will take this approach as even data that is of questionable quality may still provide relevant information to inform PrT development. To ensure that our PrT continues to provide plausible explanations for adolescent-focused low-intensity LSW, we will use an additional process for ‘quality control’. That is, we will judge the explanatory plausibility of the PrT using the criteria of consilience, simplicity and analogy [67]. Despite these measures, threats to the plausibility of our PrT may still occur, particularly if sections of it are based predominantly on data that we would judge (globally) to be of questionable ‘rigour’. In such cases, we will be explicit in highlighting and reporting these elements as limitations in project reports and future publications.

Data from all relevant full text documents will be extracted using a suitably designed and piloted standardised data collection process. We anticipate key characteristics of each included document will be extracted into an Excel spreadsheet, and that the full text of documents will be uploaded to NVivo (a qualitative data analysis software) so relevant data can be organised and coded. Coding will involve extracting relevant sections of text from included documents according to how this data can contribute to PrT development.

Step 4: Synthesising evidence

Data analysis will involve the use of a realist logic of analysis with the goal of using the data from the documents to further develop the initial PrT developed in Step 1. Data coding will be deductive (informed by the initial PrT), inductive (coming from the data within included documents) and retroductive (where inferences are made about underlying causal processes or mechanisms). Drawing on previous work [68], we will use a series of questions about the relevance and rigour of content within documents as part of our process of analysis, as set out below:

Relevance:

- Are sections of text within this document relevant to PrT development?

Rigour (judgements about trustworthiness):

- Are these data sufficiently trustworthy to warrant making changes to any aspect of the initial and emerging PrT?

Interpretation of meaning:

- If the section of text is relevant and trustworthy enough, do its contents provide data that may be interpreted as functioning as context, mechanism or outcome?

Interpretations and judgements about Context-Mechanism-Outcome-Configurations:

- For the data that has been interpreted as functioning as context, mechanism or outcome, which Context-Mechanism-Outcome-Configuration (CMOC) (partial or complete) does it belong to?
- Are there further data to inform this particular CMOC contained within this document or other documents? If so, which other documents?
- How does this particular CMOC relate to other CMOCs that have already been developed?

For peer review only - http://bmjopen.bmj.com/site/about/guidelines.xhtml

Interpretations and judgements about PrT:

- How does this (full or partial) CMOC relate to the PrT?
- Within this same document are there data which informs how the CMOC relates to the PrT? If not, are there data in other documents? Which ones?
- Considering this particular CMOC and any supporting data, does the PrT need to be changed?

Data to inform our interpretation of the relationships between contexts, mechanisms and outcomes will be sought not just within the same source, but across sources (e.g., mechanisms inferred from one document could help to explain the way contexts influenced outcomes for an intervention in another). Synthesising data from different documents is often necessary to compile CMOCs, since not all parts of the configurations can be found in the same document.

During the review, we will move iteratively between the analysis of particular examples, refinement of the PrT, and further iterative data searching to test particular theories (where needed).

During this step, we will hold our second CCEG and CEG meetings to discuss the literature, and sense check the developing PrT. The PrT and a summary of the literature will be discussed with these groups who will be asked to comment on its resonance with their perspectives and its implications for preliminary guidance. Wherever possible, we will address any gaps in the theory that persist (e.g., through additional literature searches).

Step 5: Finalising the PrT and drawing conclusions

Near the end of the review, the refined PrT will be written up and shared during the final CCEG and CEG meetings for final comments. We will seek input to ensure the outputs we produce (outlined in the section below) are useful to all stakeholders and disseminated across ‘lay’, professional and academic networks.

Ethics and dissemination

Ethical approval is not required as the realist review is secondary research.

The main outputs of this research will be an evidence-based PrT of adolescent-focused low-intensity-LSW that will inform our preliminary guidance which can be used to optimise any pre-existing practice immediately. We will share our final PrT using text, summary tables, a logic model and where appropriate youth focused information clips and/or infographics to summarise individual papers/reports and draw insights across papers/reports.

For academic, clinical, social care and educational audiences, we will produce peer-reviewed journal articles, including those detailing the process and findings of the realist review and establishing the requirements for effective adolescent-focused low-intensity LSW. For other professional audiences we will actively share our preliminary

For peer review only - http://bmjopen.bmj.com/site/about/guidelines.xhtml

guidance on the nature of good practice when delivering adolescent-focused low-intensity-LSW. This will take the form of articles and blogs.

For peer review only

For peer review only - http://bmjopen.bmj.com/site/about/guidelines.xhtml

References

1. Department for Education, *Children looked after in England including adoption, year ending 31 March 2019.*, D.f. Education, Editor. 2019: London, UK.
2. Tarren-Sweeney, M., *The mental health of children in out-of-home care.* Current Opinion in Psychiatry, 2008. **21**(4): p. 345-349.
3. Meltzer, H., et al., *The Mental Health of Young People Looked After by Local Authorities in England.* , T.T.S. Office), Editor. 2003: Londin.
4. Evans, R., et al., *Comparison of suicidal ideation, suicide attempt and suicide in children and young people in care and non-care populations: Systematic review and meta-analysis of prevalence.* Children and Youth Services Review, 2017. **82**: p. 122-129.
5. CQC, *The state of mental health services 2014 - 2017: Findings from CQCs programme of comprehensive inspections of specialist mental health services.* 2018.
6. Fang, X., et al., *The economic burden of child maltreatment in the United States and implications for prevention.* Child abuse & neglect, 2012. **36**(2): p. 156-165.
7. Conti, G., et al., *The economic cost of child maltreatment in the UK : a preliminary study*. 2017, NSPCC: London.
8. Education, D.f., *Charactersitics of young poeple who are long-term NEET*. 2018: London.
9. Luke, N., et al., *What works in preventing and treating poor mental health in looked after children?* 2014, Rees Centre: London, UK.
10. Evans, D., *Hierarchy of evidence: a framework for ranking evidence evaluating healthcare interventions.* Journal of Clinical Nursing, 2003. **12**(1): p. 77-84.
11. CEBM. *Oxford Centre for Evidence-based Medicine - Levels of Evidence (March 2009)*. 2009 [cited 2020 02.03.2020]; Available from: https://www.cebm.net/2009/06/oxford-centre-evidence-based-medicine-levels-evidence-march-2009/.
12. NICE. *Child abuse and neglect: NICE guideline [NG76]*. 2017 [cited 2020 10.02.2020]; Available from: https://www.nice.org.uk/guidance/ng76.
13. Department of Health and Department for Education, *Transforming children and young people's mental health provision: Green paper*. 2017.
14. The Education Committee, *Mental health and well-being of looked-after children*, H.o.C.E. Committee, Editor. 2016, House of Commons: London, UK.
15. Department of Health, *A Framework for mental health research*, D.o. Health, Editor. 2017: London, UK.
16. NHS. *Mental Health of Children and Young People in England, 2017*. 2018 [cited 2020 23.02.2020]; Available from: https://digital.nhs.uk/data-and-information/publications/statistical/mental-health-of-children-and-young-people-in-england/2017/2017#key-facts.
17. Action for Children, National Children's Bureau, and The Children's Society, *Turning the tide: Reversing the move to late intervention spending in children and young people's services*. 2017: London, UK.
18. Fairburn, C.G. and V. Patel, *The impact of digital technology on psychological treatments and their dissemination.* Behaviour research and therapy, 2017. **88**: p. 19-25.
19. Hollis, C., et al., *Identifying research priorities for digital technology in mental health care: results of the James Lind Alliance Priority Setting Partnership.* The Lancet Psychiatry, 2018. **5**(10): p. 845-854.
20. James, K., *Remote mental health interventions for young people: a rapid review of the evidence*. 2020, Youth Access: Championing advise and counselling: London.
21. NICE. *Common mental health problems: identification and pathways to care: Clinical guideline [CG123]* 2011 [cited 2020 22.02.2020]; Available from: https://www.nice.org.uk/guidance/cg123/chapter/Appendix-E-Glossary.
22. Kazdin, A.E. and S.M. Rabbitt, *Novel Models for Delivering Mental Health Services and Reducing the Burdens of Mental Illness.* Clinical Psychological Science, 2013. **1**(2): p. 170-191.

For peer review only

For peer review only - http://bmjopen.bmj.com/site/about/guidelines.xhtml

23. NHS, *The Improving Access to Psychological Therapies Manual*. 2019, National Collagorating Centre for Mental Health: London, UK.
24. Bellis, M.A., et al., *Life course health consequences and associated annual costs of adverse childhood experiences across Europe and North America: a systematic review and meta-analysis.* The Lancet Public Health, 2019. **4**(10): p. e517-e528.
25. Pawson, R., et al., *Realist review--a new method of systematic review designed for complex policy interventions.* J Health Serv Res Policy, 2005. **10 Suppl 1**: p. 21-34.
26. Sinclair I, et al., *The pursuit of permanence: A study of the English care system*. 2007, London, UK: Jessica Kingsley Publishers.
27. Craig, P., et al., *Developing and evaluating complex interventions: the new Medical Research Council guidance.* BMJ (Clinical research ed.), 2008. **337**: p. a1655-a1655.
28. Adoption and Children Act. *Adoption and Children Act 2002*. 2002 [cited 2020 02.03.2020]; Available from: http://www.legislation.gov.uk/ukpga/2002/38/contents.
29. Children Act. *Children Act 1989*. 1989 [cited 2020 02.03.2020]; Available from: http://www.legislation.gov.uk/ukpga/1989/41/contents.
30. Department for Education, *Guide to the Children's Homes Regulations including the quailty standards*, D.f. Education, Editor. 2015: London, UK.
31. The Fostering Services (England) Regulations. *The Fostering Services (England) Regulations 2011*. 2011 [cited 2020 02.03.2020]; Available from: http://www.legislation.gov.uk/uksi/2011/581/contents/made.
32. Ehlers, A. and D.M. Clark, *A cognitive model of posttraumatic stress disorder.* Behav Res Ther, 2000. **38**(4): p. 319-45.
33. Salmond, C.H., et al., *The nature of trauma memories in acute stress disorder in children and adolescents.* Journal of Child Psychology and Psychiatry, 2011. **52**(5): p. 560-570.
34. van Os, E.C.C., et al., *Finding Keys: A Systematic Review of Barriers and Facilitators for Refugee Children's Disclosure of Their Life Stories.* Trauma, Violence, & Abuse, 2020. **21**(2): p. 242-260.
35. Fivush, R., *Children's recollections of traumatic and nontraumatic events.* Dev Psychopathol, 1998. **10**(4): p. 699-716.
36. Mundorf, E.S. and S.C. Paivio, *Narrative quality and disturbance pre- and post-emotion-focused therapy for child abuse trauma.* J Trauma Stress, 2011. **24**(6): p. 643-50.
37. Tuval-Mashiach, R., et al., *Coping with Trauma: Narrative and Cognitive Perspectives.* Psychiatry: Interpersonal and Biological Processes, 2004. **67**(3): p. 280-293.
38. Cohen, J.A. and A.P. Mannarino, *Trauma-Focused Cognitive Behavioural Therapy for Children and Parents.* Child and Adolescent Mental Health, 2008. **13**(4): p. 158-162.
39. Foa, E.B. and M.J. Kozak, *Emotional processing of fear: Exposure to corrective information.* Psychological Bulletin, 1986. **99**(1): p. 20-35.
40. Foa, E.B. and C.P. McLean, *The Efficacy of Exposure Therapy for Anxiety-Related Disorders and Its Underlying Mechanisms: The Case of OCD and PTSD.* Annu Rev Clin Psychol, 2016. **12**: p. 1-28.
41. Rose, R., *Life Story Therapy With Traumatized Children: A Model for Practice.* . 2012, London, UK: Jessica Kingsley Publishers
42. Askelund, A.D., et al., *Positive memory specificity is associated with reduced vulnerability to depression.* Nat Hum Behav, 2019. **3**(3): p. 265-273.
43. Baynes, P., *Untold Stories: A Discussion of Life Story Work.* Adoption & Fostering, 2008. **32**(2): p. 43-49.
44. Buchanan, A., *The experience of Life Story Work: Reflections of Young People Leaving Care*. 2014, Cardiff University: Cardiff, UK. p. 181.
45. Cook-Cottone, C. and M. Beck, *A Model for Life-Story Work: Facilitating the Construction of Personal Narrative for Foster Children.* Child and Adolescent Mental Health, 2007. **12**(4): p. 193-195.

For peer review only - http://bmjopen.bmj.com/site/about/guidelines.xhtml

46. Hooley, K., L. Stokes, and H. Combes, *Life story work with looked after and adopted children: how professional training and experience determine perceptions of its value.* Adoption & Fostering, 2016. **40**(3): p. 219-233.
47. McKeown, J., A. Clarke, and J. Repper, *Life story work in health and social care: systematic literature review.* Journal of Advanced Nursing, 2006. **55**(2): p. 237-247.
48. NSPCC. *How Life Story Work works*. 2020 [cited 2020 10.02.2020]; Available from: https://learning.nspcc.org.uk/services-children-families/life-story-work/#article-top.
49. Ryan, T. and R. Walker, *Life Story Work*. 2016, London: CoramBAAF.
50. Willis, R. and S. Holland, *Life Story Work: Reflections on the Experience by Looked after Young People.* Adoption & Fostering, 2009. **33**(4): p. 44-52.
51. Wrench, K. and L. Naylor, *Life Story Work with Children Who are Fostered or Adopted: Creative Ideas and Activities*. 2013, London: Jessica Kingsley.
52. Watson, D., S. Latter, and R. Bellew, *Adopters' views on their children's life story books.* Adoption & Fostering, 2015. **39**(2): p. 119-134.
53. Watson, D.L., S. Latter, and R. Bellew, *Adopted children and young people's views on their life storybooks: The role of narrative in the formation of identities.* Children and Youth Services Review, 2015. **58**: p. 90-98.
54. Hammond, S.P., J. Young, and C. Duddy, *Life story work for children and young people with care experience: A scoping review.* Developmental Child Welfare, 2020. **2**(4): p. 293-315.
55. Hammond, S.P., N.J. Cooper, and P. Jordan, *Mental health, identity and informal education opportunities for adolescents with experience of living in state care: a role for digital storytelling.* Cambridge Journal of Education, 2021: p. 1-20.
56. Hammond, S.P., *Exploring a role for digital technology in life story work with adolescents in residential care: a discourse analysis.*, in *School of Social Work and Psychology*. 2012, University of East Anglia: Norwich.
57. Hammond, S.P. and N. Cooper, *Digital life story work: Using technology to help young people make sense of their experiences*. 2013, London: British Association of Adoption and Fostering.
58. Fahlberg, V., *A child's journey through placement. UK Edition*. 1994, London, UK: BAAF.
59. Suh, E. and L. Holmes, *A critical review of cost-effectiveness research in children's social care: What have we learnt so far?* Social Policy & Administration. **n/a**(n/a).
60. Wong, G., et al., *Realist methods in medical education research: what are they and what can they contribute?* Med Educ, 2012. **46**(1): p. 89-96.
61. Wong, G., et al., *Development of methodological guidance, publication standards and training materials for realist and meta-narrative reviews: the RAMESES (Realist And Meta-narrative Evidence Syntheses - Evolving Standards) project.* Health Serv Deliv Res, 2014. **2**(30).
62. Greenhalgh, T. and R. Peacock, *Effectiveness and efficiency of search methods in systematic reviews of complex evidence: audit of primary sources.* BMJ, 2005. **331**(7524): p. 1064-1065.
63. Booth, A., et al., *Towards a methodology for cluster searching to provide conceptual and contextual "richness" for systematic reviews of complex interventions: case study (CLUSTER).* BMC Medical Research Methodology, 2013. **13**(1): p. 118.
64. Booth, A., J. Wright, and B. S, *Scoping and searching to support realist approaches.* , in *Doing Realist Research.* , Emmel N, et al., Editors. 2018, SAGE Publications Ltd: London, UK. p. 148-65.
65. Pawson, R., *Evidence-Based Policy: A realist perspective*. 2006, London, UK: Sage.
66. Nyssen, O.P., et al., *Does therapeutic writing help people with long-term conditions? Systematic review, realist synthesis and economic considerations.* Health Technol Assess, 2016. **20**(27): p. vii-xxxvii, 1-367.
67. Wong, G., *Looking for needles in haystacks*, in *Doing Realist Research*, N. Emmel, et al., Editors. 2018, SAGE: London.

For peer review only

For peer review only - http://bmjopen.bmj.com/site/about/guidelines.xhtml

68. Papoutsi, C., et al., *Health Services and Delivery Research*, in *Interventions to improve antimicrobial prescribing of doctors in training (IMPACT): a realist review*. 2018, NIHR Journals Library

For peer review only

Author's contributions

Contributors SH conceived the study with input from GW, RH, LR, KW, EN, CD and JW. SH and EM and wrote the first draft of this protocol, and GW, CD, RH, EN, KW, JW and LR provided criticism and refinement. CD designed the search strategy. All the authors have read and approved the final manuscript.

Funding

This project is funded by the National Institute for Health Research (NIHR) Research for Patient Benefit Programme (Grant Reference Number NIHR 201963).

Disclaimer

The views expressed in this publication are those of the author(s) and not necessarily those of the NIHR or the Department of Health and Social Care.

Competing interests

SH offers consultancy services in social media and Digital Life Story Work via www.digitallifestorywork.co.uk. LR is Director of Strategy of The Care Leaders, KW is the CEO of The Fostering Network. The remaining authors declare that they have no known competing financial interests or personal relationships that could have appeared to influence the work reported in this paper.

For peer review only

For peer review only - http://bmjopen.bmj.com/site/about/guidelines.xhtml

Improving the mental health and mental health support available to adolescents with social care-experience via low-intensity life story work: A realist review protocol

Supplementary File

Search #1 Life Story Work

MEDLINE

Medline (Ovid MEDLINE® Epub Ahead of Print, In-Process & Other Non-Indexed Citations, Ovid MEDLINE® Daily and Ovid MEDLINE®) 1946 to present

Search run on 1 December 2021

	Searches	Results	Type
**1**	((life story or life history) adj2 (work or book* or resource* or tool*)).ti,ab,kw.	107	
**2**	(personal history or biograph* or autobiograph*).ti,ab,kw.	30152	
**3**	1 or 2	30262	
**4**	(teen* or youth* or adolescen* or juvenile* or young* or child* or girl? or boy?).ti,ab,kw.	2435378	
**5**	Adolescent/	2143136	
**6**	4 or 5	3811906	
**7**	looked-after.ti,ab,kw.	569	
**8**	in care.ti,ab,kw.	18400	
**9**	((foster* or social or public or state or local authority or residential or institutional or permanent or kinship or relative or substitute or out-of-home or shelter or surrogate) adj1 care*).ti,ab,kw.	19263	
**10**	(care leaver* or care-experience* or leaving care or care transition*).ti,ab,kw.	6579	
**11**	child welfare.ti,ab,kw.	5173	
**12**	(adopted or adoption or adoptive or adoptee*).ti,ab,kw.	197909	
**13**	Adoption/	4869	
**14**	Foster Home Care/	3758	
**15**	or/7-14	246890	
**16**	3 and 6 and 15	87	

For peer review only - http://bmjopen.bmj.com/site/about/guidelines.xhtml

Improving the mental health and mental health support available to adolescents with social care-experience via low-intensity life story work: A realist review protocol

Embase

Ovid Embase 1974 to Present

	Searches	Results	Type
**1**	((life story or life history) adj2 (work or book* or resource* or tool*)).ti,ab,kw.	148	
**2**	(personal history or biograph* or autobiograph*).ti,ab,kw.	25170	
**3**	1 or 2	25307	
**4**	(teen* or youth* or adolescen* or juvenile* or young* or child* or girl? or boy?).ti,ab,kw.	3050210	
**5**	adolescent/ or exp institutionalized adolescent/	1629539	
**6**	4 or 5	3924528	
**7**	looked-after.ti,ab,kw.	1002	
**8**	in care.ti,ab,kw.	26487	
**9**	((foster* or social or public or state or local authority or residential or institutional or permanent or kinship or relative or substitute or out-of-home or shelter or surrogate) adj1 care*).ti,ab,kw.	23880	
**10**	(care leaver* or care-experience* or leaving care or care transition*).ti,ab,kw.	8952	
**11**	child welfare.ti,ab,kw.	3648	
**12**	(adopted or adoption or adoptive or adoptee*).ti,ab,kw.	257330	
**13**	adoption/	18302	
**14**	foster care/ or foster child/	5008	
**15**	or/7-14	320109	
**16**	3 and 6 and 15	116	

Search run on 1 December 2021

For peer review only - http://bmjopen.bmj.com/site/about/guidelines.xhtml

Improving the mental health and mental health support available to adolescents with social care-experience via low-intensity life story work: A realist review protocol

PsycINFO

Ovid PsycINFO 1806 to Present

Search run on 1 December 2021

	Searches	Results	Type
**1**	((life story or life history) adj2 (work or book* or resource* or tool*)).ti,ab.	126	
**2**	(personal history or biograph* or autobiograph*).ti,ab.	27009	
**3**	life review/ or autobiographical memory/	5007	
**4**	or/1-3	28393	
**5**	(teen* or youth* or adolescen* or juvenile* or young* or child* or girl? or boy?).ti,ab.	1104726	
**6**	looked-after.ti,ab.	656	
**7**	in care.ti,ab.	7188	
**8**	((foster* or social or public or state or local authority or residential or institutional or permanent or kinship or relative or substitute or out-of-home or shelter or surrogate) adj1 care*).ti,ab.	19519	
**9**	(care leaver* or care-experience* or leaving care or care transition*).ti,ab.	2950	
**10**	child welfare.ti,ab.	7784	
**11**	(adopted or adoption or adoptive or adoptee*).ti,ab.	66430	
**12**	adoption (child)/ or adopted children/ or adoptive parents/	5319	
**13**	foster care/ or foster children/ or foster parents/	7544	
**14**	or/6-13	99727	
**15**	4 and 5 and 14	203	

For peer review only - http://bmjopen.bmj.com/site/about/guidelines.xhtml

Improving the mental health and mental health support available to adolescents with social care-experience via low-intensity life story work: A realist review protocol

Proquest Sociology Collection

ASSIA (Applied Social Sciences Index & Abstracts) (1987 to present); Sociological Abstracts (1952 to present); Sociology Database (1985 to present)

Search run on 1 December 2021

	Searches	Results	Type
**1**	noft(("life story" OR "life history") N/2 (work OR book* OR resource* OR tool*))	223	
**2**	noft(("personal history" OR biograph* OR autobiograph*))	30,652	
**3**	1 or 2	30,836	
**4**	noft((teen* OR youth* OR adolescen* OR juvenile* OR young* OR child* OR boy* OR girl*))	776,851	
**5**	noft("looked after" OR "looked-after")	1,477	
**6**	noft("in care")	8,932	
**7**	noft((foster* OR social OR public OR state OR "local authority" OR residential OR institutional OR permanent OR kinship OR relative OR substitute OR "out of home" OR out-of-home OR shelter OR surrogate) N/1 care*)	49,937	
**8**	noft("care leaver" OR "care experience*" OR care-experience* OR "leaving care" OR "care transition*")	3,219	
**9**	noft("child welfare")	27,228	
**10**	noft(adopted OR adoption OR adoptive OR adoptee*)	35,937	
**11**	5 or 6 or 7 or 8 or 9 or 10	110774	
**12**	3 and 4 and 12	372	
**Full**	(noft(("life story" OR "life history") NEAR/2 (work OR book* OR resource* OR tool*)) OR noft(("personal history" OR biograph* OR autobiograph*))) AND (noft("looked after" OR "looked-after") OR noft("in care") OR noft((foster* OR social OR public OR state OR "local authority" OR residential OR institutional OR permanent OR kinship OR relative OR substitute OR "out of home" OR out-of-home OR shelter OR surrogate) NEAR/1 care*) OR noft("care leaver" OR "care experience*" OR care-experience* OR "leaving care" OR "care transition*") OR noft("child welfare") OR noft(adopted OR adoption OR adoptive OR adoptee*)) AND noft((teen* OR youth* OR adolescen* OR juvenile* OR young* OR child* OR boy* OR girl*))		

For peer review only - http://bmjopen.bmj.com/site/about/guidelines.xhtml

Improving the mental health and mental health support available to adolescents with social care-experience via low-intensity life story work: A realist review protocol

CINAHL

Ebscohost CINAHL (Cumulative Index to Nursing and Allied Health Literature) (Start date unknown)

Search run 1 December 2021

	Searches	Results	Type
**S1**	TI ("life story" OR "life history") N2 (work OR book* OR resource* OR tool*) OR AB ("life story" OR "life history") N2 (work OR book* OR resource* OR tool*)	135	
**S2**	TI ("personal history" OR biograph* OR autobiograph*) OR AB ("personal history" OR biograph* OR autobiograph*)	6148	
**S3**	(MH "Life History Review") OR (MH "Autobiographical Memory")	1592	
**S4**	S1 OR S2 OR S3	7675	
**S5**	TI teen* OR youth* OR adolescen* OR juvenile* OR young* OR child* OR girl* OR boy* OR AB teen* OR youth* OR adolescen* OR juvenile* OR young* OR child* OR girl* OR boy*	1475230	
**S6**	(MH "Adolescence")	564984	
**S7**	S5 OR S6	1475230	
**S8**	TI "looked after" OR looked-after OR AB "looked after" OR looked-after	718	
**S9**	TI "in care" OR AB "in care"	929827	
**S10**	TI (foster* OR social OR public OR state OR "local authority" OR residential OR institutional OR permanent OR kinship OR relative OR substitute OR "out of home" OR out-of-home OR shelter OR surrogate) N1 care* OR AB (foster* OR social OR public OR state OR "local authority" OR residential OR institutional OR permanent OR kinship OR relative OR substitute OR "out of home" OR out-of-home OR shelter OR surrogate) N1 care*	31046	
**S11**	TI ("care leaver" OR "care experience*" OR care-experience* OR "leaving care" OR "care transition*") OR AB ("care leaver" OR "care experience*" OR care-experience* OR "leaving care" OR "care transition*")	5417	
**S12**	TI "child welfare" OR AB "child welfare"	4202	
**S13**	TI adopted or adoption or adoptive or adoptee* OR AB adopted or adoption or adoptive or adoptee*	57750	
**S14**	(MH "Adoption") OR (MH "Child, Adopted") OR (MH "Adoptive Parents")	4085	
**S15**	(MH "Foster Home Care") OR (MH "Foster Parents") OR (MH "Child, Foster")	6372	
**S16**	S8 OR S9 OR S10 OR S11 OR S12 OR S13 OR S14 OR S15	9 75840	
**S17**	S4 AND S7 AND S16	359	

For peer review only - http://bmjopen.bmj.com/site/about/guidelines.xhtml

Improving the mental health and mental health support available to adolescents with social care-experience via low-intensity life story work: A realist review protocol

CDAS

Ebscohost CDAS (Child Development and Adolescent Studies) (Start date unknown)

Search run 1 December 2021

	Searches	Results	Type
**S1**	TI ("life story" OR "life history") N2 (work OR book* OR resource* OR tool*) OR AB ("life story" OR "life history") N2 (work OR book* OR resource* OR tool*)	29	
**S2**	TI ("personal history" OR biograph* OR autobiograph*) OR AB ("personal history" OR biograph* OR autobiograph*)	1442	
**S3**	S1 OR S2	1471	
**S4**	TI "looked after" OR looked-after OR AB "looked after" OR looked-after	696	
**S5**	TI "in care" OR AB "in care"	32026	
**S6**	TI (foster* OR social OR public OR state OR "local authority" OR residential OR institutional OR permanent OR kinship OR relative OR substitute OR "out of home" OR out-of-home OR shelter OR surrogate) N1 care* OR AB (foster* OR social OR public OR state OR "local authority" OR residential OR institutional OR permanent OR kinship OR relative OR substitute OR "out of home" OR out-of-home OR shelter OR surrogate) N1 care*	7379	
**S7**	TI ("care leaver" OR "care experience*" OR care-experience* OR "leaving care" OR "care transition*") OR AB ("care leaver" OR "care experience*" OR care-experience* OR "leaving care" OR "care transition*")	651	
**S8**	TI "child welfare" OR AB "child welfare"	5715	
**S9**	TI adopted or adoption or adoptive or adoptee* OR AB adopted or adoption or adoptive or adoptee*	6798	
**S10**	S4 OR S5 OR S6 OR S7 OR S8 OR S9	41463	
**S11**	S3 AND S10	86	

NB Search string describing adolescents removed due to population focus in this database

For peer review only - http://bmjopen.bmj.com/site/about/guidelines.xhtml

Improving the mental health and mental health support available to adolescents with social care-experience via low-intensity life story work: A realist review protocol

Web of Science

Clarivate Web of Science Core Collection (SCI-EXPANDED and SSCI indexes) 1900 to present

Search run 1 December 2021

	Searches	Results	Type
**1**	TS=((("life story" OR "life history") NEAR/2 (work OR book* OR resource* OR tool*)))	449	
**2**	TS=((("personal history" OR biograph* OR autobiograph*)))	41850	
**3**	1 OR 2	41850	
**4**	TS=(((teen* OR youth* OR adolescen* OR juvenile* OR young* OR child* OR boy* OR girl*)))	3066217	
**5**	TS=(("looked after" OR "looked-after"))	768	
**6**	TS=(("in care"))	16889	
**7**	TS=((foster* OR social OR public OR state OR "local authority" OR residential OR institutional OR permanent OR kinship OR relative OR substitute OR "out of home" OR out-of-home OR shelter OR surrogate) NEAR/1 care*)	43040	
**8**	TS=("care leaver" OR "care experience*" OR care-experience* OR "leaving care" OR "care transition*")	6615	
**9**	TS=("child welfare")	8348	
**10**	TS=(adopted OR adoption OR adoptive OR adoptee*)	655351	
**11**	5 OR 6 OR 7 OR 8 OR 9 OR 10	721923	
**12**	3 AND 4 AND 11	233	

For peer review only - http://bmjopen.bmj.com/site/about/guidelines.xhtml

Improving the mental health and mental health support available to adolescents with social care-experience via low-intensity life story work: A realist review protocol

Social Care Online

SCIE Social Care Online 1980 to present

Search run 3 February 2022

	Searches	Results	Type
**1**	“life story” OR “life history”		
**2**	teen OR youth OR adolescent OR juvenile OR young OR child OR girl OR boy		
**3**	“looked after” OR “foster care” OR “social care” OR “public care” OR “state care” OR “local authority care” OR “residential care” OR "institutional care" OR "permanent care" OR "kinship care" OR "relative care" OR "substitute care" OR "out of home care" OR "shelter care" OR adopted OR adoption OR adoptive OR adoptee		
**4**	(1 AND 2 AND 3)	58	
	NB Search strategies were adapted for simpler search interface; word variations included automatically		

For peer review only

For peer review only - http://bmjopen.bmj.com/site/about/guidelines.xhtml

Improving the mental health and mental health support available to adolescents with social care-experience via low-intensity life story work: A realist review protocol

Search #2 Low intensity mental health interventions

MEDLINE

Medline (Ovid MEDLINE® Epub Ahead of Print, In-Process & Other Non-Indexed Citations, Ovid MEDLINE® Daily and Ovid MEDLINE®) 1946 to present

Search run on 2 February 2022

	Searches	Results	Type
**1**	(teen* or youth* or adolescen* or juvenile*).ti,ab,kw.	486326	
**2**	Adolescent/	2155772	
**3**	1 or 2	2326981	
**4**	looked-after.ti,ab,kw.	585	
**5**	((foster* or social or public or state or local authority or residential or institutional or permanent or kinship or relative or substitute or out-of-home or shelter or surrogate) adj1 care*).ti,ab,kw.	19561	
**6**	(care leaver* or care-experience* or leaving care or care transition*).ti,ab,kw.	6715	
**7**	child welfare.ti,ab,kw.	5217	
**8**	Adoption/	4875	
**9**	Foster Home Care/	3770	
**10**	or/4-9	37119	
**11**	((adopted or adoption or adoptive or adoptee* or in-care or "in care") adj3 (teen* or youth* or adolescen* or juvenile* or young* or child* or girl? or boy?)).ti,ab,kw.	3708	
**12**	10 or 11	38990	
**13**	((low-intensity or minimal or brief or online or internet or tele* or mobile* or e-health or m-health or virtual) adj2 (intervention* or program* or service* or package* or training* or therap* or treatment*)).ti,ab,kw.	48784	
**14**	Therapy, Computer-Assisted/	6949	
**15**	Computer-Assisted Instruction/	12318	
**16**	Telemedicine/	32176	
**17**	or/13-16	92385	
**18**	(lay-therapy or lay-therapist* or lay-worker* or lay-person* or lay-people).ti,ab,kw.	2143	
**19**	(para-professional* or non-specialist* or non-clinician* or health worker* or support worker*).ti,ab,kw.	24797	
**20**	((unqualified or unregistered or volunt*) adj2 (therap* or worker* or coach* or facilitator* or practitioner*)).ti,ab,kw.	1337	
**21**	Community Health Workers/	6092	
**22**	or/18-21	30823	
**23**	(self-help or self-manage*).ti,ab,kw.	31158	
**24**	Self Care/	35033	
**25**	or/23-24	57185	
**26**	((group* or peer*) adj2 (intervention* or program* or service* or package* or training* or therap* or treatment)).ti,ab,kw.	228457	

For peer review only - http://bmjopen.bmj.com/site/about/guidelines.xhtml

Improving the mental health and mental health support available to adolescents with social care-experience via low-intensity life story work: A realist review protocol

**27**	Self-Help Groups/	9435
**28**	or/26-27	236909
**29**	17 or 22 or 25 or 28	398896
**30**	(mental health or wellbeing or well-being).ti,ab,kw.	280239
**31**	mental health/ or exp mental disorders/	1382592
**32**	Quality of Life/	232462
**33**	or/30-32	1732433
**34**	3 and 12 and 29 and 33	160
**35**	limit 34 to english language	151

For peer review only

For peer review only - http://bmjopen.bmj.com/site/about/guidelines.xhtml

Improving the mental health and mental health support available to adolescents with social care-experience via low-intensity life story work: A realist review protocol

Embase

Ovid Embase 1974 to Present

Search run on 2 February 2022

	Searches	Results	Type
**1**	(teen* or youth* or adolescen* or juvenile*).ti,ab,kw.	601244	
**2**	adolescent/ or institutionalized adolescent/	1644483	
**3**	1 or 2	1868684	
**4**	looked-after.ti,ab,kw.	1012	
**5**	((foster* or social or public or state or local authority or residential or institutional or permanent or kinship or relative or substitute or out-of-home or shelter or surrogate) adj1 care*).ti,ab,kw.	24293	
**6**	(care leaver* or care-experience* or leaving care or care transition*).ti,ab,kw.	9146	
**7**	child welfare.ti,ab,kw.	3695	
**8**	adoption/	18546	
**9**	foster care/ or foster child/	5037	
**10**	or/4-9	56946	
**11**	((adopted or adoption or adoptive or adoptee* or in-care or "in care") adj3 (teen* or youth* or adolescen* or juvenile* or young* or child* or girl? or boy?)).ti,ab,kw.	4485	
**12**	10 or 11	59432	
**13**	((low-intensity or minimal or brief or online or internet or tele* or mobile* or e-health or m-health or virtual) adj2 (intervention* or program* or service* or package* or training* or therap* or treatment*)).ti,ab,kw.	65233	
**14**	computer assisted therapy/	4801	
**15**	Computer-Assisted Instruction/	81963	
**16**	telemedicine/ or exp teleconsultation/ or exp telemonitoring/ or exp telepsychiatry/ or exp telepsychology/ or exp teletherapy/ or exp video consultation/	51669	
**17**	or/13-16	193129	
**18**	(lay-therapy or lay-therapist* or lay-worker* or lay-person* or lay-people).ti,ab,kw.	2836	
**19**	(para-professional* or non-specialist* or non-clinician* or health worker* or support worker*).ti,ab,kw.	29728	
**20**	((unqualified or unregistered or volunt*) adj2 (therap* or worker* or coach* or facilitator* or practitioner*)).ti,ab,kw.	1615	
**21**	voluntary worker/ or exp volunteer/	64329	
**22**	lay health worker/ or mental health care personnel/ or health auxiliary/	11904	
**23**	or/18-22	103668	
**24**	(self-help or self-manage*).ti,ab,kw.	43320	

For peer review only - http://bmjopen.bmj.com/site/about/guidelines.xhtml

Improving the mental health and mental health support available to adolescents with social care-experience via low-intensity life story work: A realist review protocol

**25**	self care/ or self help/	80302
**26**	or/24-25	94122
**27**	((group* or peer*) adj2 (intervention* or program* or service* or package* or training* or therap* or treatment)).ti,ab,kw.	333041
**28**	group therapy/	19771
**29**	or/27-28	343422
**30**	17 or 23 or 26 or 29	706680
**31**	(mental health or wellbeing or well-being).ti,ab,kw.	349720
**32**	exp mental health/ or exp mental disease/	2482186
**33**	quality of life/ or exp wellbeing/	614976
**34**	or/31-33	3026040
**35**	3 and 12 and 30 and 34	283
**36**	limit 35 to english language	274

For peer review only

For peer review only - http://bmjopen.bmj.com/site/about/guidelines.xhtml

Improving the mental health and mental health support available to adolescents with social care-experience via low-intensity life story work: A realist review protocol

PsycINFO

Ovid PsycINFO 1806 to Present

Search run on 2 February 2022

	Searches	Results	Type
**1**	(teen* or youth* or adolescen* or juvenile*).ti,ab.	344737	
**2**	looked-after.ti,ab.	662	
**3**	((foster* or social or public or state or local authority or residential or institutional or permanent or kinship or relative or substitute or out-of-home or shelter or surrogate) adj1 care*).ti,ab.	19672	
**4**	(care leaver* or care-experience* or leaving care or care transition*).ti,ab.	2980	
**5**	child welfare.ti,ab.	7836	
**6**	adoption (child)/ or adopted children/ or adoptive parents/	5337	
**7**	foster care/ or foster children/ or foster parents/	7582	
**8**	or/2-7	34369	
**9**	((adopted or adoption or adoptive or adoptee* or in-care or "in care") adj3 (teen* or youth* or adolescen* or juvenile* or young* or child* or girl? or boy?)).ti,ab.	5753	
**10**	8 or 9	36284	
**11**	((low-intensity or minimal or brief or online or internet or tele* or mobile* or e-health or m-health or virtual) adj2 (intervention* or program* or service* or package* or training* or therap* or treatment*)).ti,ab.	28828	
**12**	exp computer assisted therapy/	11642	
**13**	exp computer assisted instruction/	21848	
**14**	or/11-13	57608	
**15**	(lay-therapy or lay-therapist* or lay-worker* or lay-person* or lay-people).ti,ab.	1793	
**16**	(para-professional* or non-specialist* or non-clinician* or health worker* or support worker*).ti,ab.	7045	
**17**	((unqualified or unregistered or volunt*) adj2 (therap* or worker* or coach* or facilitator* or practitioner*)).ti,ab.	746	
**18**	volunteers/	5507	
**19**	or/15-18	14762	
**20**	(self-help or self-manage*).ti,ab.	19273	
**21**	self-care/	3097	
**22**	self-help techniques/	4398	
**23**	or/20-22	23360	

For peer review only - http://bmjopen.bmj.com/site/about/guidelines.xhtml

Improving the mental health and mental health support available to adolescents with social care-experience via low-intensity life story work: A realist review protocol

**24**	((group* or peer*) adj2 (intervention* or program* or service* or package* or training* or therap* or treatment)).ti,ab.	75638
**25**	group psychotherapy/	20529
**26**	support groups/	4449
**27**	or/24-26	86800
**28**	14 or 19 or 23 or 27	173409
**29**	(mental health or wellbeing or well-being).ti,ab.	289304
**30**	exp mental health/	77446
**31**	exp mental disorders/	919736
**32**	well being/ or exp spiritual well being/	51717
**33**	quality of life/	44133
**34**	or/29-33	1164452
**35**	1 and 10 and 28 and 34	137
**36**	limit 35 to english language	130

For peer review only

For peer review only - http://bmjopen.bmj.com/site/about/guidelines.xhtml

Improving the mental health and mental health support available to adolescents with social care-experience via low-intensity life story work: A realist review protocol

Proquest Sociology Collection

ASSIA (Applied Social Sciences Index & Abstracts) (1987 to present); Sociological Abstracts (1952 to present); Sociology Database (1985 to present)

Search run on 2 February 2022

	Searches	Results	Type
**Full String**	(noft((teen* OR youth* OR adolescen* OR juvenile*)) AND (noft("looked after" OR "looked-after") OR noft((foster* OR social OR public OR state OR "local authority" OR residential OR institutional OR permanent OR kinship OR relative OR substitute OR "out of home" OR out-of-home OR shelter OR surrogate) N/1 care*) OR noft("care leaver" OR "care experience*" OR care-experience* OR "leaving care" OR "care transition*") OR noft("child welfare") OR noft(((adopted or adoption or adoptive or adoptee* or in-care or "in care") N/3 (teen* or youth* or adolescen* or juvenile* or young* or child* or girl? or boy?)))) AND (noft((low-intensity or minimal or brief or online or internet or tele* or mobile* or e-health or m-health or virtual) N/2 (intervention* or program* or service* or package* or training* or therap* or treatment*)) OR noft((lay-therapy or lay-therapist* or lay-worker* or lay-person* or lay-people)) OR noft((para-professional* or non-specialist* or non-clinician* or ("health worker" OR "health workers") or ("support worker" OR "support workers"))) OR noft((unqualified OR unregistered OR volunt*) NEAR/2 (therap* OR worker* OR coach* OR facilitator* OR practitioner*)) OR noft((self-help OR self-manage*)) OR noft((group* or peer*) N/2 (intervention* or program* or service* or package* or training* or therap* or treatment)))) AND noft("mental health" or wellbeing or well-being)	249	
	(Filter: English language)		

For peer review only - http://bmjopen.bmj.com/site/about/guidelines.xhtml

Improving the mental health and mental health support available to adolescents with social care-experience via low-intensity life story work: A realist review protocol

CINAHL

Ebscohost CINAHL (Cumulative Index to Nursing and Allied Health Literature) (Start date unknown)

Search run 2 February 2022

	Searches	Results	Type
**S32**	S25 AND S30	471	
**S31**	S25 AND S30	477	
**S30**	S26 OR S27 OR S28 OR S29	835,613	
**S29**	(MH "Quality of Life")	127,552	
**S28**	(MH "Mental Disorders+")	611,172	
**S27**	(MH "Mental Health")	46,077	
**S26**	TI ("mental health" OR wellbeing OR well-being) OR AB ("mental health" or wellbeing or well-being)	182,035	
**S25**	S12 AND S24	1,451	
**S24**	S13 OR S14 OR S15 OR S16 OR S17 OR S18 OR S19 OR S20 OR S21 OR S22 OR S23	216,784	
**S23**	(MH "Support Groups")	11,056	
**S22**	TI (((group* or peer*) N2 (intervention* or program* or service* or package* or training* or therap* or treatment))) OR AB (((group* or peer*) N2 (intervention* or program* or service* or package* or training* or therap* or treatment)))	99,368	
**S21**	(MH "Self Care")	42,907	
**S20**	TI ("self help" OR self-help OR "self manage*" OR self-manage*) OR AB ("self help" OR self-help OR "self manage*" OR self-manage*)	20,838	
**S19**	(MH "Community Health Workers")	4,008	
**S18**	TI (((unqualified or unregistered or volunt*) N2 (therap* or worker* or coach* or facilitator* or practitioner*))) OR AB (((unqualified or unregistered or volunt*) N2 (therap* or worker* or coach* or facilitator* or practitioner*)))	962	
**S17**	TI (("lay therapy" or lay-therapy or "lay therapist*" or lay-therapist* or "lay worker*" or lay-worker* or "lay person*" or lay-person* or "lay people" or lay-people)) AND AB (("lay therapy" or lay-therapy or "lay therapist*" or lay-	82	

For peer review only - http://bmjopen.bmj.com/site/about/guidelines.xhtml

Improving the mental health and mental health support available to adolescents with social care-experience via low-intensity life story work: A realist review protocol

	therapist* or "lay worker*" or lay-worker* or "lay person*" or lay-person* or "lay people" or lay-people))	
**S16**	(MH "Telemedicine") OR (MH "Remote Consultation") OR (MH "Telepsychiatry")	17,115
**S15**	(MH "Computer Assisted Instruction")	8,174
**S14**	(MH "Therapy, Computer Assisted")	5,457
**S13**	TI (((low-intensity or "low intensity" or minimal or brief or online or internet or tele* or mobile* or e-health or m-health or virtual) N2 (intervention* or program* or service* or package* or training* or therap* or treatment*))) OR AB (((low-intensity or "ow intensity" or minimal or brief or online or internet or tele* or mobile* or e-health or m-health or virtual) N2 (intervention* or program* or service* or package* or training* or therap* or treatment*)))	34,674
**S12**	S10 AND S11	19,442
**S11**	S3 OR S4 OR S5 OR S6 OR S7 OR S8 OR S9	77,416
**S10**	S1 OR S2	626,344
**S9**	(MH "Foster Home Care") OR (MH "Foster Parents") OR (MH "Child, Foster")	6,425
**S8**	(MH "Adoption") OR (MH "Child, Adopted") OR (MH "Adoptive Parents")	4,102
**S7**	TI (((adopted OR adoption OR adoptive OR adoptee* OR "in-care" OR "in care") N2 (teen* OR youth* OR adolescen* OR juvenile* or young* OR child*))) OR AB (((adopted OR adoption OR adoptive OR adoptee* OR "in-care" OR "in care") N2 (teen* OR youth* OR adolescen* OR juvenile* OR young* OR child*)))	37,133
**S6**	TI "child welfare" OR AB "child welfare"	4,278
**S5**	TI ("care leaver" OR "care experience*" OR care-experience* OR "leaving care" OR "care transition*") OR AB ("care leaver" OR "care experience*" OR care-experience* OR "leaving care" OR "care transition*")	5,533
**S4**	TI (foster* OR social OR public OR state OR "local authority" OR residential OR institutional OR permanent OR kinship OR relative OR substitute OR "out of home" OR out-of-home OR shelter OR surrogate) N1 care* OR AB (foster* OR social OR public OR state OR "local authority" OR residential OR institutional OR permanent OR kinship OR relative OR substitute OR "out of home" OR out-of-home OR shelter OR surrogate) N1 care*	31,528
**S3**	TI "looked after" OR looked-after OR AB "looked after" OR looked-after	723
**S2**	(MH ""Adolescence"")	569,286
**S1**	TI (teen* OR youth* OR adolescen* OR juvenile*) OR AB (teen* OR youth* OR adolescen* OR juvenile*)	209,502

For peer review only - http://bmjopen.bmj.com/site/about/guidelines.xhtml

Improving the mental health and mental health support available to adolescents with social care-experience via low-intensity life story work: A realist review protocol

For peer review only

For peer review only - http://bmjopen.bmj.com/site/about/guidelines.xhtml

Improving the mental health and mental health support available to adolescents with social care-experience via low-intensity life story work: A realist review protocol

CDAS

Ebscohost CDAS (Child Development and Adolescent Studies) (Start date unknown)

Search run 3 February 2022

	Searches	Results	Type
**S1**	TI (teen* OR youth* OR adolescen* OR juvenile*) OR AB (teen* OR youth* OR adolescen* OR juvenile*)	99,382	
**S2**	TI "looked after" OR looked-after OR AB "looked after" OR looked-after	698	
**S3**	TI (foster* OR social OR public OR state OR "local authority" OR residential OR institutional OR permanent OR kinship OR relative OR substitute OR "out of home" OR out-of-home OR shelter OR surrogate) N1 care* OR AB (foster* OR social OR public OR state OR "local authority" OR residential OR institutional OR permanent OR kinship OR relative OR substitute OR "out of home" OR out-of-home OR shelter OR surrogate) N1 care*	7434	
**S4**	TI ("care leaver" OR "care experience*" OR care-experience* OR "leaving care" OR "care transition*") OR AB ("care leaver" OR "care experience*" OR care-experience* OR "leaving care" OR "care transition*")	656	
**S5**	TI "child welfare" OR AB "child welfare"	5752	
**S6**	TI (((adopted OR adoption OR adoptive OR adoptee* OR "in-care" OR "in care") N2 (teen* OR youth* OR adolescen* OR juvenile* or young* OR child*))) OR AB (((adopted OR adoption OR adoptive OR adoptee* OR "in-care" OR "in care") N2 (teen* OR youth* OR adolescen* OR juvenile* OR young* OR child*)))	14908	
**S7**	TI (((low-intensity or "low intensity" or minimal or brief or online or internet or tele* or mobile* or e-health or m-health or virtual) N2 (intervention* or program* or service* or package* or training* or therap* or treatment*))) OR AB (((low-intensity or "ow intensity" or minimal or brief or online or internet or tele* or mobile* or e-health or m-health or virtual) N2 (intervention* or program* or service* or package* or training* or therap* or treatment*)))	2481	
**S8**	TI (("lay therapy" or lay-therapy or "lay therapist*" or lay-therapist* or "lay worker*" or lay-worker* or "lay person*" or lay-person* or "lay people" or lay-people)) AND AB (("lay therapy" or lay-therapy or "lay therapist*" or lay-therapist* or "lay worker*" or lay-worker* or "lay	5	

For peer review only - http://bmjopen.bmj.com/site/about/guidelines.xhtml

Improving the mental health and mental health support available to adolescents with social care-experience via low-intensity life story work: A realist review protocol

	person*" or lay-person* or "lay people" or lay-people))	
**S9**	TI (((unqualified or unregistered or volunt*) N2 (therap* or worker* or coach* or facilitator* or practitioner*))) OR AB (((unqualified or unregistered or volunt*) N2 (therap* or worker* or coach* or facilitator* or practitioner*)))	78
**S10**	TI ("self help" OR self-help OR "self manage*" OR self-manage*) OR AB ("self help" OR self-help OR "self manage*" OR self-manage*)	804
**S11**	TI (((group* or peer*) N2 (intervention* or program* or service* or package* or training* or therap* or treatment))) OR AB (((group* or peer*) N2 (intervention* or program* or service* or package* or training* or therap* or treatment)))	6798
**S12**	TI ("mental health" OR wellbeing OR well-being) OR AB ("mental health" or wellbeing or well-being)	24106
**S13**	S2 OR S3 OR S4 OR S5 OR S6	22614
**S14**	S7 OR S8 OR S9 OR S10 OR S11	9795
**S15**	S1 AND S12 AND S13 AND S14	61

For peer review only

For peer review only - http://bmjopen.bmj.com/site/about/guidelines.xhtml

Improving the mental health and mental health support available to adolescents with social care-experience via low-intensity life story work: A realist review protocol

Web of Science

Clarivate Web of Science Core Collection (SCI-EXPANDED and SSCI indexes) 1900 to present

Search run 2 February 2022

	Searches	Results	Type
1	TS=(((teen* OR youth* OR adolescen* OR juvenile*)))	757523	
2	TS=(("looked after" OR "looked-after"))	776	
3	TS=((foster* OR social OR public OR state OR "local authority" OR residential OR institutional OR permanent OR kinship OR relative OR substitute OR "out of home" OR out-of-home OR shelter OR surrogate) NEAR/1 care*)	43729	
4	TS=("care leaver" OR "care experience*" OR care-experience* OR "leaving care" OR "care transition*")	6760	
5	TS=("child welfare")	8425	
6	TS=(((adopted or adoption or adoptive or adoptee* or "in-care" or "in care") NEAR/3 (teen* or youth* or adolescen* or juvenile* or young* or child* or girl? or boy?)))	7120	
7	or/2-6	61902	
8	TS=(((low-intensity or minimal or brief or online or internet or tele* or mobile* or e-health or m-health or virtual) NEAR/2 (intervention* or program* or service* or package* or training* or therap* or treatment*)))	90429	
9	TS=((lay-therapy or lay-therapist* or lay-worker* or lay-person* or lay-people))	2561	
10	TS=((para-professional* or non-specialist* or non-clinician* or health worker* or support worker*))	120315	
11	TS=(((unqualified or unregistered or volunt*) NEAR/2 (therap* or worker* or coach* or facilitator* or practitioner*)))	1938	
12	TS=(self-help or self-manage*)	41426	
13	TS=((group* or peer*) NEAR/2 (intervention* or program* or service* or package* or training* or therap* or treatment))	263661	
14	#13 OR #12 OR #11 OR #10 OR #8 OR #9	499546	
15	TS=((mental health or wellbeing or well-being))	409139	
16	#15 and #14 and #7 and #1		
17	limit to english language		

For peer review only - http://bmjopen.bmj.com/site/about/guidelines.xhtml

Improving the mental health and mental health support available to adolescents with social care-experience via low-intensity life story work: A realist review protocol

Social Care Online

SCIE Social Care Online 1980 to present

Search run 3 February 2022

	Searches	Results	Type
**1**	teen or youth or adolescent or juvenile		
**2**	looked after OR "foster care" OR "social care" OR "public care" OR "state care" OR "local authority care" OR "residential care" OR "institutional care" OR "permanent care" OR "kinship care" OR "relative care" OR "substitute care" OR "out of home care" OR "shelter care" OR adopted OR adoption OR adoptive OR adoptee		
**3**	*low intensity OR minimal OR brief OR online OR digital OR internet OR tele OR mobile OR e-health OR m-health OR virtual*		
**4**	mental health OR wellbeing OR "well being"		
**5**	1 and 2 and 3 and 4	29	
**1**	teen or youth or adolescent or juvenile		
**2**	looked after OR "foster care" OR "social care" OR "public care" OR "state care" OR "local authority care" OR "residential care" OR "institutional care" OR "permanent care" OR "kinship care" OR "relative care" OR "substitute care" OR "out of home care" OR "shelter care" OR adopted OR adoption OR adoptive OR adoptee		
**3**	*"lay therapy" OR "lay therapist" OR "lay worker" OR "lay person" OR "lay people" OR "paraprofessional" OR "non-specialist" OR "non-clinician" OR "health worker" OR coach OR facilitator OR practitioner OR unqualified OR unregistered OR volunteer*		
**4**	mental health OR wellbeing OR "well being"		
**5**	1 and 2 and 3 and 4	7	
**1**	teen or youth or adolescent or juvenile		
**2**	looked after OR "foster care" OR "social care" OR "public care" OR "state care" OR "local authority care" OR "residential care" OR "institutional care" OR "permanent care" OR "kinship care" OR "relative care" OR "substitute care" OR "out of home care" OR "shelter care" OR adopted OR adoption OR adoptive OR adoptee		
**3**	*"self care" OR "self help" OR "self management" OR group OR peer*		
**4**	mental health OR wellbeing OR "well being"		
**5**	1 and 2 and 3 and 4	21	
	NB *search strategies adapted for simpler search interface and run in three separate queries*		

For peer review only - http://bmjopen.bmj.com/site/about/guidelines.xhtml

Improving the mental health and mental health support available to adolescents with social care-experience via low-intensity life story work: A realist review protocol

For peer review only

For peer review only - http://bmjopen.bmj.com/site/about/guidelines.xhtml
